# Carbon nanotube and graphene fiber artificial muscles

**DOI:** 10.1039/c9na00038k

**Published:** 2019-10-30

**Authors:** Javad Foroughi, Geoffrey Spinks

**Affiliations:** School of Electrical, Computer and Telecommunications Engineering, Faculty of Engineering and Information Sciences, Intelligent Polymer Research Institute, University of Wollongong Australia NSW 2522 Australia Foroughi@uow.edu.au

## Abstract

Actuator materials capable of producing a rotational or tensile motion are rare and, yet, rotary systems are extensively utilized in mechanical systems like electric motors, pumps, turbines and compressors. Rotating elements of such machines can be rather complex and, therefore, difficult to miniaturize. Rotating action at the microscale, or even nanoscale, would benefit from the direct generation of torsion from an actuator material. Herein we discuss the advantages of using carbon nanotube (CNT) yarns and/or graphene (G) fibers as novel artificial muscles that have the ability to be driven by the electrochemical charging of helically wound multiwall carbon nanotubes or graphene fibers as well as elements in the ambient environment such as moisture to generate such rotational action. The torsional strain, torque, speed and lifetime have been evaluated under various electrochemical conditions to provide insight into the actuation mechanism and performance. Here the most recent advances in artificial muscles based on sheath-run artificial muscles (SRAMs) are reviewed. Finally, the rotating motion of the CNT yarn actuator and the humidity-responsive twisted graphene fibers have been coupled to a mixer for use in a prototype microfluidic system, moisture management and a humidity switch respectively.

## Abstract 2

Recent advances in artificial muscles based on twisted and coiled carbon nanotube yarns and graphene fibers.

## Graphical abstract

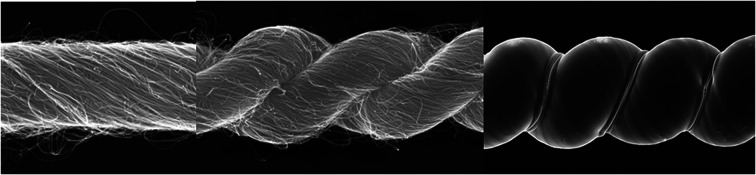

## 1. Introduction

Actuating materials, or ‘artificial muscles’, continue to attract a great deal of interest because of the need to develop compact and lightweight motor systems for robotics, micro-fluidics, medical prosthetics and micro-machines. Carbon nanotube actuators have been known for some time^1,2^ but performance limitations have restricted their applications. The recent discoveries that carbon nanotube yarns generate giant torsional (rotating) actuation^3^ and the conversion of torsion to large lengthwise tensile actuation in coiled fibers^4^ have dramatically increased the performance and application areas for carbon nanotube actuators.

The properties of carbon nanotubes combined with the extreme twist insertion in carbon nanotube yarns are the source of the large torsional and tensile actuation strokes. Remarkable performance has been obtained for tensile and torsional carbon nanotube hybrid yarn muscles,^5–8^ whose actuation is driven by the volume change of a guest that is within a twisted or coiled carbon nanotube yarn host. During thermally powered contraction, coiled hybrid muscles can provide 29 times the work and generate 85 times the power of the same weight of natural muscle.^4^ Carbon nanotube hybrid yarn artificial muscles are made by inserting twist, or both twist and coiling, into a guest-filled CNT yarn. Muscles that are twisted (but not coiled), which are called twisted muscles, are mainly useful for torsional actuation. Extremely high inserted twist results in coiled muscles that can deliver tensile strokes exceeding those of nature's skeletal muscles.^4^ More recently a new generation of artificial muscles was reported based on core–sheath hybrid carbon nanotube yarns or commercially available yarns. Since the dimensional and modulus changes of the sheath drive torsional and tensile actuation, they were called “sheath-run artificial muscles” (SRAMs).^9^

Inspired by the initial work with twisted CNT based yarns, researchers have also developed high performance torsional and tensile actuators using other materials. For example, actuator materials based on highly twisted wet-spun graphene fibers and hybrid carbon nanotube/graphene fibers have been developed recently.^10–16^ Even metallic nanowires from niobium have been formed into twisted yarns that deliver fast and large torsional actuation at low input voltages. Shape memory alloys from nickel and titanium have also been formed into twisted two-ply fibers that generate a reversible torsional stroke of 16° mm^−1^ of muscle length at a peak rotation speed of up to 10, 500 revolutions per minute.^8,17,18^

In this work we review the current state-of-the-art torsional actuators made from twisted CNTs and graphene fibers. The methods used to fabricate CNTs and graphene fibers are described along with those configurations used to demonstrate and optimise both torsional and tensile actuation. Finally we explore the current understanding of the fundamental actuation mechanisms in these systems.

## 2. Preparation of carbon nanotube yarns

**Fig. 1:**
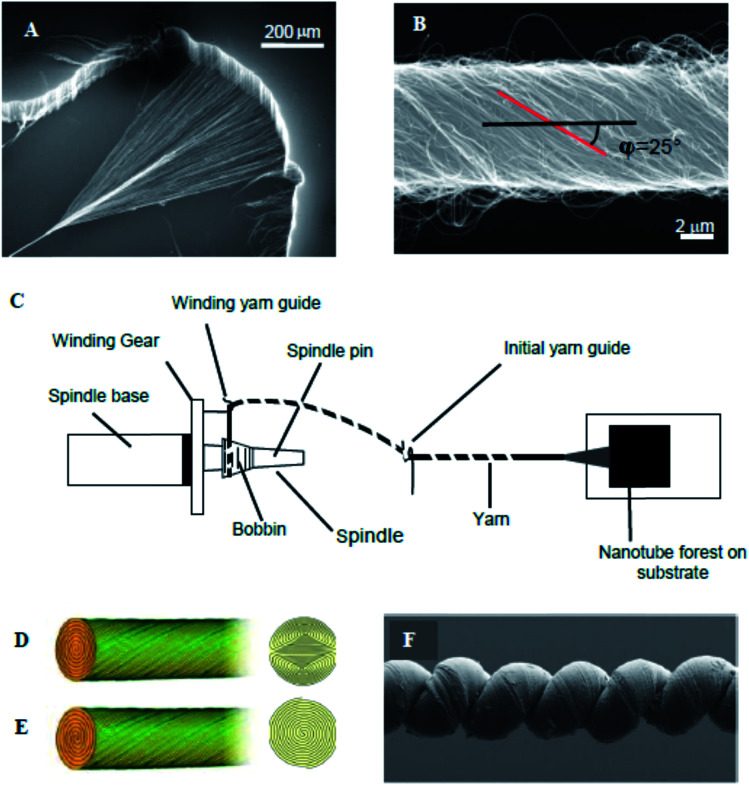
(A) SEM micrograph of a MWNT forest and (B) SEM micrograph of a carbon nanotube yarn that was symmetrically twist spun from a MWNT forest. Schematic diagram of spinning of a MWNT yarn from a multi-walled carbon nanotube forest (C); and idealized cross-sections for Fermat (D) and dual-Archimedean (E) scroll structures spun symmetrically and highly asymmetrically, respectively, from a carbon nanotube forest. (F) SEM micrograph of a coiled, wax-infiltrated hybrid MWNT yarn. These figures have been reproduced from ref. 3 and 21 with permission from AAAS and The Royal Society of Chemistry respectively.

High performance carbon nanotube yarns can be prepared from carbon multiwalled nanotube (MWNT) forests grown by chemical vapor deposition (CVD) using acetylene (C_2_H_2_) gas as the carbon precursor. The growth substrate was a silicon wafer coated with a 1–3 nm thick iron catalyst layer by e-beam evaporation. This substrate was loaded into a 3-inch diameter quartz tube furnace that had been heated to 700 °C in a mixture of 750 sccm Ar and 100 sccm H_2_. After a 5 minute residence time at oven temperature, a nanotube forest was grown in 2 to 5 minutes after introduction of 50 sccm of C_2_H_2_ to this gas mixture. The presently investigated yarns were drawn from ∼400 μm high forests of carbon MWNTs during the symmetric insertion of twist [Fig. 1A].^19,20^ In the method now described a key property that enables spinning directly from the forest is the degree of intertwining between neighbouring tubes. When nanotubes are plucked from the forest, their neighbours will follow, as shown.^19,20^ Transmission and scanning electron microscopy (TEM and SEM) images indicate that the MWNTs have an outer diameter of ∼12 nm, contain ∼9 walls, and form large bundles that are ∼400 μm high.^3^ Thermogravimetric analysis shows that the content of non-combustible material in the drawn nanotubes is below 1 wt%, which places an upper limit on the amount of residual catalyst.

Small and large diameter yarns were fabricated directly from the MWNT forests [Fig. 1B]. Small diameter yarns were made by symmetrical twist insertion during sheet draw from a forest to give a Fermat scroll geometry [Fig. 1D].^20^ Alternatively, the sheet drawn from the forest can be suspended between two rigid end supports. Twisting the end supports in opposite directions gives dual-Archimedean scrolls [Fig. 1E]. The yarn diameter could be conveniently varied from ∼10 μm to ∼30 μm by changing the drawn forest width from ∼0.5 cm to ∼5 cm. Much larger diameter dual-Archimedean yarns were typically fabricated by first stacking 20 to 40 MWNT sheets between the rigid rods and then inserting twist using an electric motor.^4,22^ Tension was applied to the MWNT sheets during twisting, for example by vertically suspending a weight that was constrained to prevent rotation.

The amount of inserted twist per final yarn length (*T*) and the final yarn diameter (*d*) are important parameters, which determine the bias angle (*α*) between the nanotube orientation on the yarn surface and the yarn major axis. For Fermat yarns, the theoretical relationship *α* = tan^−1^(π*dT*) is consistent with SEM observations of the yarn surface, despite the complex nature of the realized yarn structure, which contains stochastic elements due to such processes as sheet pleating during twist insertion. A strictly topological equation to predict *α* from only *d* and *T* does not exist for a dual-Archimedean scroll, since the number of turns inserted by plying two Archimedean scrolls into a dual-Archimedean scroll (*versus* the initial number of turns that provide twist in each Archimedean scroll) is a consequence of yarn energetics.

Coiled yarns (Fig. 1F) were typically fabricated from non-coiled, twist-spun yarns under constant load by inserting additional twist until the yarn contracted to 30–40% of its original length. For a dual-Archimedean yarn of 100 μm diameter and made under 4 g load by twist insertion in a stack of 40 co-oriented, 9 mm wide, 15 cm long sheets, coiling started at ∼580 turns and the yarn was completely coiled after ∼620 turns. Complete coiling produced an ∼60% contraction in yarn length.^4^

Some studies have used guest materials incorporated into the porous MWNT yarn host. The guest typically provides volumetric expansion upon heating or swelling, with this expansion being constrained in the direction of nanotube orientation, which in turn acts to amplify torsion, as discussed below. Examples demonstrated to date include water-sensitive poly(ethylene glycol), hydrogen-absorbing palladium, and thermo-sensitive polydiacetylene and paraffin, with most attention to date focussed on the latter. Various commercially obtained waxes (like those used for canning and candles) were successfully used as thermo-sensitive guest materials contained within the MWNT yarn host. Most studies have used Sigma-Aldrich 411671 wax, which comprises a mixture of alkanes, fully melts at ∼83 °C, expands by ∼20% between 30 and 90 °C during solid-state transitions and melting, and provides ∼10% additional volume expansion between 90 and 210 °C.^4^

**Fig. 2:**
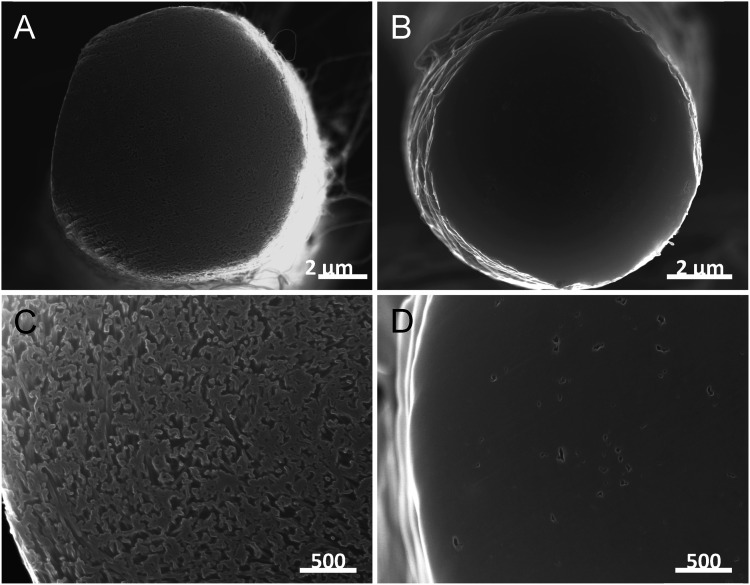
The SEM images of the cross-section of a twisted carbon nanotube yarn before (A and C) and after (B and D) wax infiltration. Pores shown in the high magnification image of the neat yarn (C) are not visible in the corresponding image of the wax hybrid yarn (D). This figure has been reproduced from ref. 4 with permission from AAAS.

MWNT yarns were typically infiltrated with paraffin wax using the “hot wire method”, wherein a two-end-tethered, twist-spun yarn, under constant tensile load was electrically heated to above the melting point of the paraffin wax and then brought into contact with a small amount of solid paraffin. Upon touching the heated yarn with flakes of solid paraffin or droplets of molten paraffin, the paraffin quickly spread through and absorbed into the yarn.^4^ Care must be taken to avoid adding too much paraffin, since excess paraffin on the yarn surface degraded actuation. In such cases, heating the yarn to above the evaporation temperature of the paraffin (∼233 °C) could remove the surface excess paraffin. Another wax infiltration method, which was used for all Fermat yarns that were directly twist spun during forest draw, is to slowly immerse a two-end-tethered, as-spun yarn into melted paraffin (∼0.1 cm s^−1^) under constant tensile load (∼10% of the failure stress). The SEM micrographs of Fig. 2 show that the porosity of the neat yarn has been largely eliminated by wax infiltration using the above slow immersion method.

## 3. Torsional actuation

The first observation of torsional actuation in MWNT yarns was an accidental discovery that occurred during routine electrochemical characterisation of yarn capacitance. A single piece of neat MWNT yarn immersed in liquid electrolyte and clamped at one end that was also electrically connected to a potentiostat was observed to slowly rotate as the electrochemical potential applied to the yarn was changed.^3^ The direction of yarn rotation depended on the voltage scan direction. The first systematic studies of the phenomenon also used electrochemical stimulation and led to the discovery of giant torsional actuation strokes of up to 250° mm^−1^ rotation per actuating yarn length with speeds of ∼600 revolutions per minute. These large torsional actuation strokes were at least 1000 times higher than for previously reported torsional actuators based on shape memory alloys and ceramic piezoelectric materials.^3^ These initial studies also demonstrated that the torsional actuation was a result of yarn volume increase that accompanies electrochemical charging. Subsequent work used non-electrochemical means to generate yarn volume change such as simple absorption of solvent or, more usefully, the electro-thermal expansion of an incorporated guest material. The latter enabled fully dry torsional actuation systems that did not require the electrolyte, counter electrode and packaging needed for electrochemically driven actuators.^3^

### 3.1 Optimal configurations for torsional actuation

**Fig. 3:**
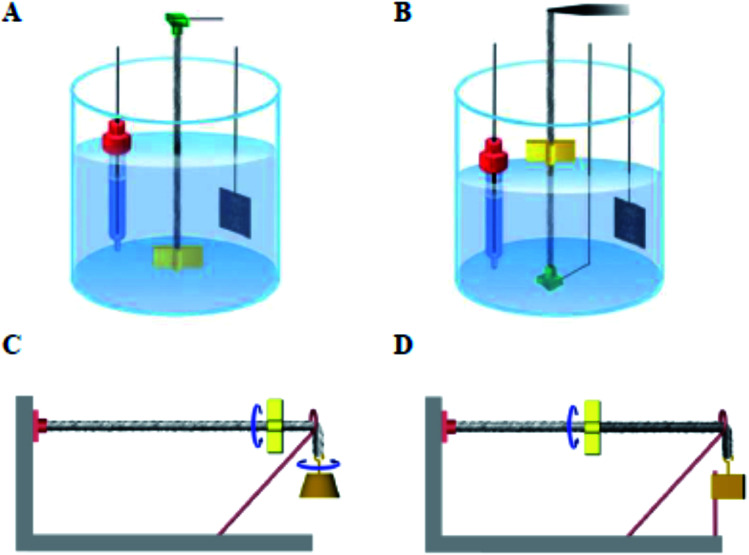
Illustration of electrochemical (A and B) and electrothermal (C and D) configurations used for characterizing torsional actuation or the combination of torsional and tensile actuation. The electrochemical configuration using a liquid electrolyte bath where the reference electrode, actuating MWNT yarn electrode, and Pt mesh counter electrode are from left to right. (A) A one-end-tethered yarn configuration in which a paddle, located at the yarn end, rotates in the electrolyte. (B) A two-end tethered configuration for simultaneously measuring torsional and tensile actuation, in which the top yarn support is a force/distance transducer that maintains constant tensile force on the yarn and measures the axial length change as the paddle rotates in air (in other cases the electrolyte level was raised to submerge the paddle). (C) A one-end-tethered and horizontally suspended hybrid yarn where an attached weight provides tension and is free to rotate. (D) A two-end-tethered configuration where the tensioning weight is prevented from rotating. In both (C) and (D) the tensioning weight can move vertically in response to yarn tensile actuation. This figure has been reproduced from ref. 3 with permission from AAAS.

**Fig. 4:**
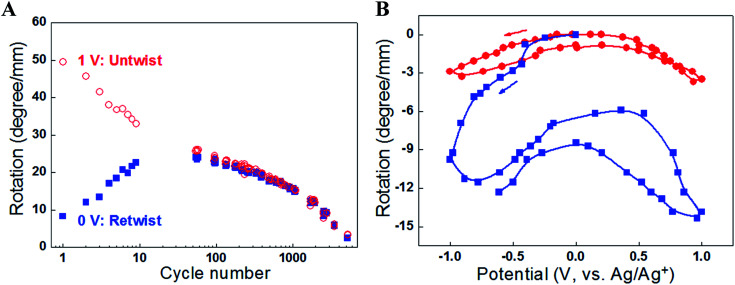
(A) The torsional rotation of a paddle attached to the free end of a one-end-tethered, 65 mm long MWNT yarn during cycling between +1 V and 0 V (*vs.* Ag/AgCl) in 1 M NaClO_4_ aqueous electrolyte. The red open circles indicate the torsional rotations on actuation to +1 V (which are in the untwist direction), while the blue solid squares correspond to the reverse rotations (twist direction) induced when the voltage was returned to 0 V. The yarn has a diameter of 12.2 μm and a bias angle of 40°. (B) Torsional rotation angle of a paddle attached to a MWNT yarn and cycled between −1 V and +1 V in 0.2 M TBAPF_6_/acetonitrile electrolyte at 50 mV s^−1^. Red circles are for a paddle attached to the middle of a two-end-tethered yarn (47 mm long, with 24 mm actuating length) and blue squares are for a paddle attached to the bottom end of a 20 mm long, one-end-tethered yarn that is fully immersed in electrolyte. The yarn diameter is 12 μm and the bias angle is *α* = 42°. This figure has been reproduced from ref. 3 with permission from AAAS.

Initial investigations into torsional actuation focussed on developing an appropriate testing methodology for reproducibly and accurately measuring torsional strokes. Specific experimental details are provided in the published literature and a summary of the main issues is provided here. The simplest test method involved vertically suspending the sample with a paddle attached at its free end to allow observation of rotation by video recording or other means of optical detection. In the electrochemical system the sample was fully immersed in a liquid electrolyte bath that also contained a counter electrode and reference electrode [Fig. 3A]. The wax-filled hybrid yarns were suspended horizontally [Fig. 3C] and tensioned by attaching a weight *via* a stiff cord that passed over a pulley. The results obtained from these methods highlighted a problem of reversibility in these ‘one-end-tethered’ torsional actuators. As illustrated in Fig. 4A, the amount of untwist occurring during electrochemical charging at +1 V (*vs.* Ag/AgCl) exceeded the degree of re-twist occurring during discharge to 0 V. During a cyclic sweep of voltage the incomplete reversibility of the torsional actuation is observed as an offset between the initial and final paddle rotation angle [Fig. 4B]. Interestingly, the electrochemical torsional actuation became fully reversible following a series of ‘training cycles’ [Fig. 4A]. The degree of untwist declined steadily during all cycles. In contrast, the amount of retwist initially increased to eventually match the amount of untwist. Subsequent cycles saw an equivalent decline in both untwist and retwist.

**Fig. 5:**
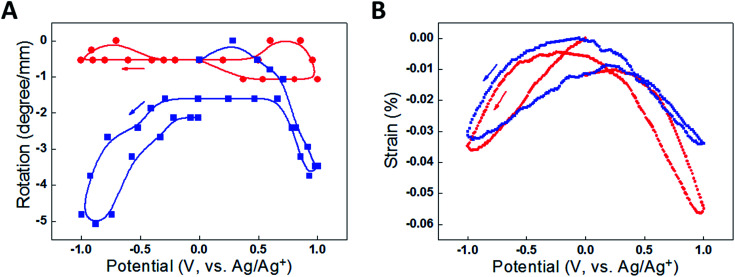
Data comparing actuation for a fully immersed (red) and half immersed (blue) two-end tethered yarn of total length 36 mm cycled between +1 V and −1 V in 0.2 M TBAPF_6_/acetonitrile electrolyte at 50 mV s^−1^. (A) Paddle rotation per actuating yarn length as a function of potential, where the lines are a guide to the eye, and (B) length direction actuation strain per actuating yarn length as a function of potential. The yarn has a diameter of 13 μm and a bias angle of 36°. This figure has been reproduced from ref. 3 with permission from AAAS.

The problems of reversibility and cycle stability were solved by employing a ‘two-end-tethered’ configuration with an asymmetric configuration to permit rotation. Simply clamping the sample at both ends and activating the entire length (such as by electrochemically charging a sample fully immersed in electrolyte) produced no rotation because of symmetry cancelling [Fig. 5A]. Instead, if only part of the sample was activated (such as occurs when only part of the sample was immersed in electrolyte) then the asymmetry allows rotation. As shown in Fig. 5A, the half-immersed, two-end-tethered electrochemically charged sample showed fully reversible rotation of a paddle attached at the yarn mid-point. The non-actuating portion of the sample now acts as a ‘return spring’ to aid reversibility. The amplitude of the torsional stroke was reduced, however, compared with the free-end rotation in the one-end-tethered, fully-immersed case, due to the mechanical constraint imposed by the return spring. A detailed description of the torsion mechanics of these configurations is provided in Section 3.2. An additional advantage of the two-end-tethered system is the ability to accurately position the rotating element, as is required in most applications.

An alternative solution to aid reversibility is to use a guest material that remains solid during activation. Indeed, heating and cooling the paraffin-filled MWNT yarns to temperatures below the wax melting point provided reversible torsional actuation without the need for a return spring. Other examples of reversible torsional actuation have been reported in electrochemically driven MWNT yarn torsional muscles using solid state electrolytes^23^ and elastomer filled MWNT yarns.^24^ These latter systems have used a heterochiral two-end-tethered configuration wherein two oppositely handed yarn segments are used to break symmetry. The two-end-tethering allows accurate positioning of the paddle attached at the junction between the two yarn segments. No loss of torsional stroke occurs since now both segments are active. The solid guest material provides the necessary elasticity to ensure torsional reversibility.

### 3.2 Torsion mechanics analysis of torsional actuation

This analysis describes the mechanics of torsional actuation for yarns tethered at either one end or at two ends. The analysis explains the effect of paddle position and relative lengths of actuating and non-actuating yarns on the reported torsional stroke. The goal is to provide a unifying method for comparing torsional strokes measured using different configurations and with different length samples.

**Fig. 6:**
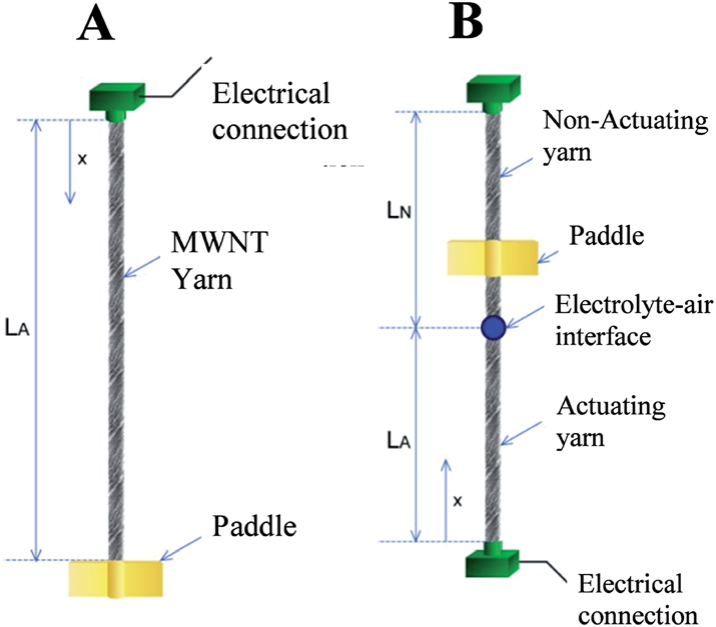
Illustration of a yarn tethered at one end (A) and at two ends (B). In the latter case the yarn is only partially actuating, as occurs when an electrochemically activated yarn is partially immersed in electrolyte, or is made from two pieces of yarn that are electrically insulated, or if an expandable guest is added to only one part of a hybrid yarn. For all of these two-end-tethered configurations, there is an actuating yarn length *L*_A_ and non-actuating (or return spring) length *L*_N_. This figure has been reproduced from ref. 3 with permission from AAAS.

The analysis first considers a vertically hanging yarn of length *L* that is clamped at the top end and is free to rotate at the opposite end, where a paddle is attached. The rotation (in degrees or radians) of the free end of a torsional shaft with respect to the clamped end is *ϕ*(*L*) = *T*/*S*, where *T* is the torque applied to the free end and *S* is the torsional rigidity of the shaft. The actuating yarn generates a rotation per yarn length of Δ*θ* when activated while tethered at only one end. The rotation at any distance *x* from the tethered end to the free yarn end [Fig. 6A] is simply *ϕ*(*x*) = *x*Δ*θ*.

The general case of a yarn tethered at both ends is next considered. Rotation is measured using a paddle attached to the yarn at a distance *x* from one end, as shown in Fig. 6B. Clamping the yarn at both ends restricts rotation at all points along the yarn by generating equal and opposite residual torques in the actuating and non-actuating segments. The net rotation, *ϕ*(*x*) occurring at any point *x* along the yarn is given by:

Rotation in the actuating yarn segment:1a
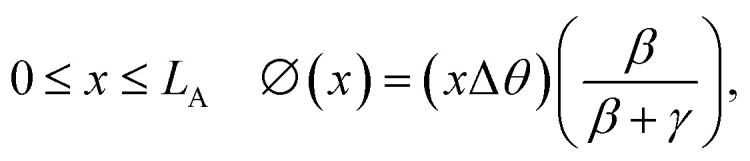


Rotation in the non-actuating yarn segment:1b
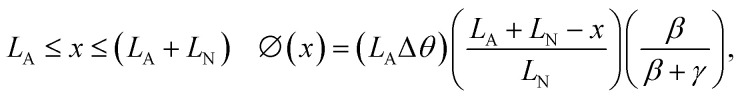
where *β* and *γ* are the ratios of the torsional modulus and lengths, respectively, of the actuating yarn and non-actuating yarn segments.

**Fig. 7:**
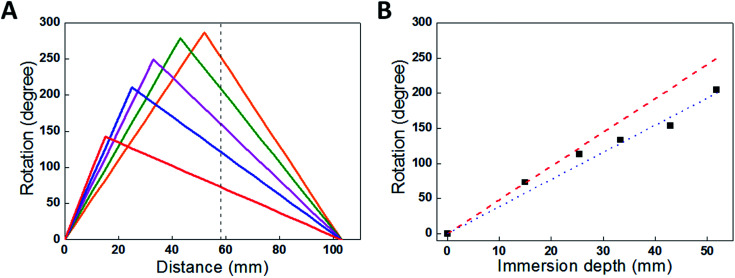
(A) Calculated rotation angle as a function of position along a 103 mm yarn that is immersed to various depths in the electrolyte: 15 mm, 25 mm, 33 mm, 43 mm and 52 mm. The dashed line indicates the paddle position used to obtain experimental results. (B) Rotation measured at position *x* = 58 mm (the paddle location) for a 103 mm long yarn immersed in progressively increased electrolyte depths (which provides the length of the actuating yarn). Red dashed and blue dotted lines are the calculated rotations from eqn (1a) and (1b) using *β* = 1 and *β* = 0.7, respectively. The black squares are measurement results. This figure has been reproduced from ref. 3 with permission from AAAS.

For comparison with the predictions of eqn (1a) and (1b), the torsional actuation for an electrochemically activated, two-end-tethered yarn was measured as a function of immersion depth in electrolyte. For yarns immersed to different depths in the electrolyte, Fig. 7A plots calculated yarn rotation angles (for a potential change from 0 V to −1 V) as a function of distance along the yarn for *β* = 1 and Δ*θ* = −11° mm^−1^, the latter being the fit parameter for comparison with experimental results. The maximum rotation angle is at the junction between actuating (*i.e.*, immersed) and non-actuating yarn segments, while the dotted vertical line is the fixed location of the paddle in experiments. The paddle rotations shown in Fig. 7B are for the intersections between this line and the calculated rotation curves. The dotted line assumes a torsional modulus ratio (*β*) of unity, while the dashed line uses a torsional modulus ratio of *β* = 0.7, which is within the measured range. The fitted Δ*θ* = −11° mm^−1^ provides good agreement between the calculated and observed paddle rotation when the appropriate torsional modulus ratio was used.

The above analysis shows that the paddle rotation produced by yarn activation depends upon the type of yarn clamping, the relative lengths of actuating and non-actuating yarns, their relative torsional moduli, and the location of the paddle. The above described Δ*θ* is a fundamental torsional parameter that is directly related to the mechanism of torsional actuation. This parameter can be used to derive torsional rotation for yarns in various configurations. For example: *ϕ*_f_ = *L*_o_Δ*θ* for the free-end rotation for one-end tethering and 
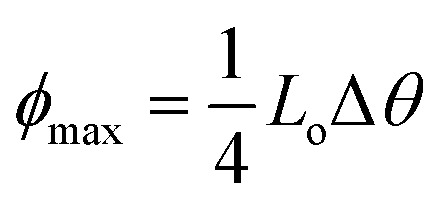
 for the maximum rotation in the two-end tethered configuration (which occurs at the mid-point of a half-active yarn), where *L*_o_ is the total yarn length and *β* is approximated to be one.

### 3.3 Torsional actuation performance

A striking feature of the torsional actuation observed in MWNT yarns is the very large and fast torsional strokes achievable. The observed torsional rotation strokes in carbon nanotube yarns and subsequently reported nanofiber yarns are more than 1000 times larger than previously reported for materials that actuate to provide torsion. Prior to the discovery of torsional actuation in carbon nanotube yarns, the largest torsional strokes were from hollow-rod torsional actuators based on shape memory alloys (SMAs) and piezoelectric ceramics which generated torsional rotations of 0.15° mm^−1^ (ref. 25) and 0.008° mm^−1^,^26,27^ respectively.

#### 3.3.1 Electrochemical torsional actuation

**Fig. 8:**
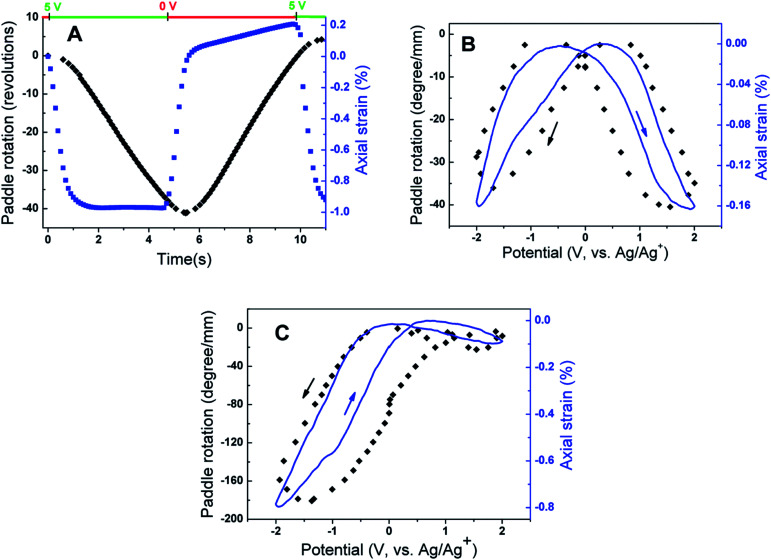
Actuation results for electrochemically activated two-end tethered MWNT yarns. (A) Torsional rotation (black) and axial length actuation (blue) *versus* time for a 120 mm long MWNT yarn (*d* = 12 μm and *α* = 40°) that is half immersed in 0.2 M TBAPF_6_ in acetonitrile and pulsed to +5 V (*vs.* Ag/Ag^+^ reference) and then to 0 V for about 5 seconds each. (B and C) Paddle rotation (normalised to the length of the actuating yarn) during a cyclic potential scan at 50 mV s^−1^. Two immersed MWNT yarns of 18 mm length (*d* = 12 μm and *α* = 42°) are separated by an insulating fiber at the midpoint, where the paddle is located, and only the lower yarn segment is actuated. The electrolytes are (B) 0.1 M BMP·TFSI in propylene carbonate electrolyte and (C) 0.2 M TBA·PF_6_ in acetonitrile electrolyte. Arrows indicate the voltage scan direction. This figure has been reproduced from ref. 3 with permission from AAAS.

The first demonstration of torsional actuation in nanofiber yarns used electrochemical charging of MWNT yarns. A highest reported stroke of over 41 full turns (15 000°) was produced [Fig. 8A] by rapid electrochemical charging of a 12 μm diameter yarn in an organic electrolyte. The maximum torsional rotation rate was 590 rotations per minute, which was approximately maintained for over 4 seconds and 30 full rotations. A similar long-sustained high rotation rate was achieved in the opposite direction on return of the electrode potential to 0 V. Since the measured yarn twist inserted during spinning was 20 000 turns per m, and 6 cm of the 12 cm total yarn length was in the electrolyte, the torsional actuation amounted to 3.4% of the twist inserted in the actuating yarn length.

Slow voltage scan experiments [Fig. 8B and C] show that the torsional stroke increases with voltage deviation on either side of the potential of zero charge (approximately 0 V on the reference potential scale indicated). We postulate that torsional actuation arises from the pressure generated by change in the relative concentrations of ions drawn into the yarn volume to compensate for injected electronic charge on the nanotubes (and associated changes in solvating species, especially when the ion size is small). Volume increases upon electrochemical charging of high-surface-area carbon supercapacitor electrodes, including single wall carbon nanotube sheets, have been previously related to changes in ion/solvent concentrations within the porous electrodes.^28^ Support for this pressure-driven actuation mechanism is found in Fig. 8, where the torsional actuation depends upon the size of the electrolyte ion used to compensate electronic charge. Specifically, the much larger actuation during reduction than for oxidation in Fig. 8B for the tetrabutylammonium hexafluorophosphate (TBAPF_6_) electrolyte reflects the larger unsolvated van der Waals volume^29^ for the tetrabutylammonium cation (293 Å^3^) than for the hexafluorophosphate anion (69 Å^3^). Likewise, the almost identical oxidative and reductive actuator strokes [Fig. 8C] for 1-butyl-1-methylpyrrolidinium bis(trifluoromethanesulfonyl)imide (BMP·TFSI) electrolyte agree with the similar van der Waals volumes of the anion (147 Å^3^) and cation (167 Å^3^) in the salt.

Rapid pulse electrochemical charging was used to assess the torque and power generated by the twisted MWNT yarns. The initial paddle acceleration in Fig. 8A was *a* = 50 rad s^−2^ (9000° s^−2^). Since the moment of inertia (*I*) of the paddle was ∼2 × 10^−10^ kg m^2^, the weight of the actuating yarn was 5.4 μg, and yarn charging occurred during the initial acceleration period, and the maximum start-up torque (*τ*) was at least *τ* = *Ia* = 10 nN m, which is 1.85 N m per kilogram of actuating yarn weight. The kinetic energy generated in the paddle 
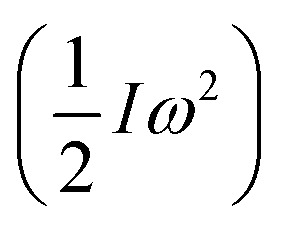
, normalized by the 1.2 s time needed to accelerate the initially stationary paddle to maximum speed and the weight of the actuating yarn, provides a peak power output of 71 W kg^−1^. The specific torque and power densities are similar to those achieved by large commercial electric motors, ranging from 2.5 to 6 N m kg^−1^ and up to 300 W kg^−1^, respectively.^30^

These figures of merit for torque generation and power density for nanotube yarns do not incorporate the electrolyte, counter electrode, connector, and packaging masses, which reduce the gravimetric power output of optimized batteries and supercapacitors by typically 3 to 8 fold compared to that based on the mass of the working electrode. Packaging to contain liquid electrolytes may also inhibit actuation, so efforts have been made to construct MWNT yarn actuators using solid electrolytes. In one study,^23^ complete two-electrode torsional and tensile actuators were constructed from two ply MWNT yarns infiltrated with poly(vinylidene fluoride-co-hexafluoropropylene), containing tetraethylammonium tetrafluoroborate with propylene carbonate solid gel electrolyte. Electrochemical charging produced 53° per mm one-end-tethered torsional actuation. Maximum rotation speeds of ∼2330 revolutions per minute were measured and peak specific torques were up to 0.067 N m kg^−1^.

#### 3.3.2 Torsional actuation in hybrid MWNT yarns

**Fig. 9:**
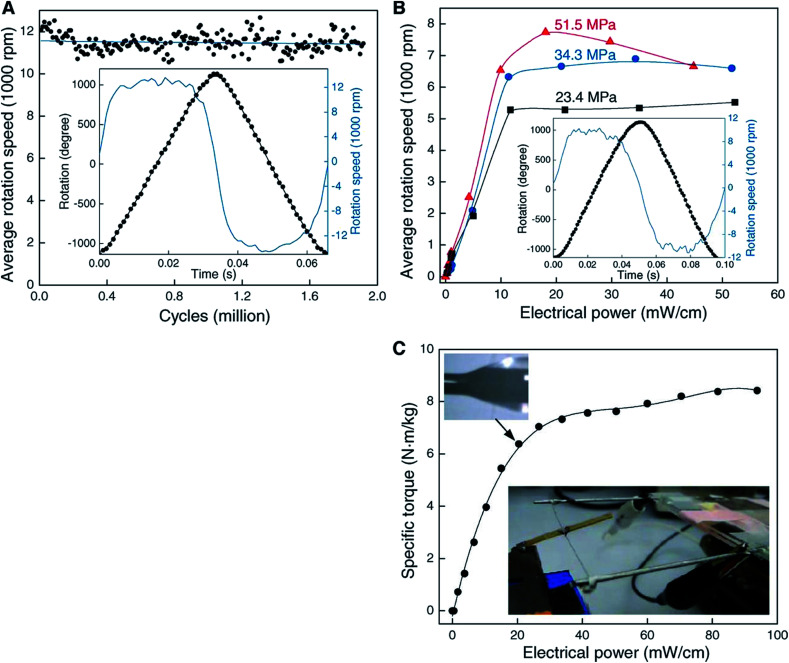
Torsional actuation for two-end-tethered, wax-infiltrated hybrid MWNT yarns. (A) Average rotation rate *versus* cycle number for a 6.9 cm long, 10 μm diameter and ∼22 000 turns per m twist yarn that was half infiltrated with wax and when excited by a 15 Hz, 40 V cm^−1^, square wave voltage using a 50% duty cycle and 41 MPa applied load. Each point on the graph is the average speed over 120 cycles. Inset: rotation angle and rotation speed *versus* time for one complete cycle. The average rotation speed was ∼11 500 revolutions/minute over nearly 2 million cycles. (B) Average rotation rate *versus* applied electrical power for different tensile loads when using the yarn in (A) and a heavier paddle. Inset: rotation angle and speed *versus* time for 51.5 MPa load. The average speed was 7600 revolutions per minute. (C) Static torque *versus* applied electrical power for a 100 μm diameter, 6.4 cm long, fully-infiltrated, heterochiral, dual-Archimedean yarn having ∼3000 turns per m of inserted twist per stack length. This figure has been reproduced from ref. 4 with permission from AAAS.

Hybrid yarns with expandable guests are alternative solid-state torsional actuators and are easier to construct than their electrochemical counterparts. Fast rotations of up to 11 500 rotations per minute were obtained using electrothermal heating of half-wax-filled hybrid yarns [Fig. 9A], albeit with smaller attached paddles (and a correspondingly lower moment of inertia) than were used in the electrochemical experiments. As expected, torsional strokes increased with increasing applied input electrical power used for yarn heating [Fig. 9B]. Maximum actuation strokes in two-end-tethered configurations of ∼30° per mm of actuator length were developed in the hybrid yarns compared with up to 250° per mm for electrochemically activated neat yarns operated in liquid electrolytes. Wax-filled niobium yarns generated only 12° per mm of actuator length^31^ probably as a result of a smaller maximum twist insertion that could occur without fibre breakage in comparison with the MWNT yarns.

The wax-filled hybrid MWNT yarns gave very stable torsional actuation with a long cycle life. Very fast, highly reversible torsional actuation was demonstrated for two million cycles for a 10 μm diameter, two-end-tethered, half-wax-infiltrated MWNT yarn [Fig. 9A]. Testing ceased after two million heating/cooling cycles wherein fully reversible untwist and retwist of a full cycle stroke of ∼58° per mm of actuator length occurred. Negligible degradation in cycle amplitude or rotation speed was detected throughout the 2 million cycles.

The maximum specific torque was directly measured by rotating an attached paddle against a force transducer. Using a 100 μm diameter yarn (∼10 times thicker than typically used) the maximum specific torque was 8.42 N m kg^−1^. This value is five times higher than demonstrated for smaller diameter electrochemically driven nanotube yarns and slightly higher than for large electric motors (up to 6 N m kg^−1^).

#### 3.3.3 Absorption driven torsional actuation in MWNT yarns

**Fig. 10:**
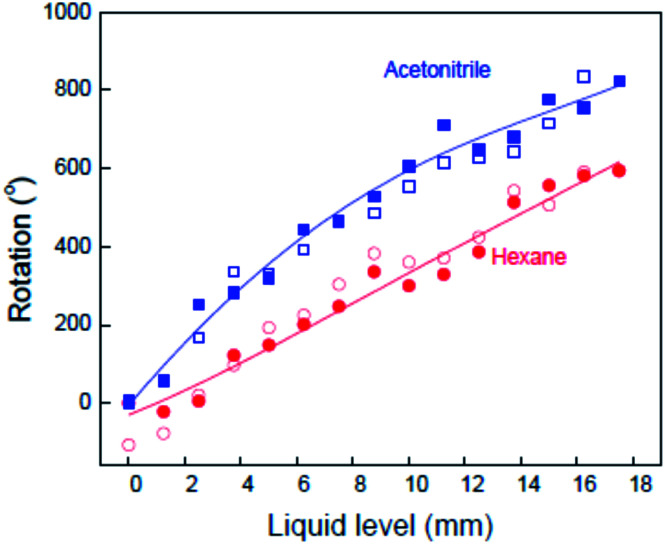
The observed dependence of the paddle rotation angle on the yarn immersion depth in acetonitrile and in hexane for an 8 μm diameter, neat MWNT yarn having 25 000 turns per m of inserted twist. Closed and open symbols are for liquid filling and removal, respectively. The lines are a guide to the eye. This figure has been reproduced from ref. 4 with permission from AAAS.

Reversible torsional actuation could also be achieved by absorption and desorption of small molecules. For example, a MWNT yarn containing a 60 nm thick palladium layer on the nanotube bundles caused 1.5 paddle rotations within ∼6 s on exposure to hydrogen gas (one-end-tethered). The rotation was fully reversed on a similar time scale during repeated cycling between hydrogen exposure and vacuum. Liquid absorption and desorption can also drive actuation, as shown in Fig. 10, where torsional actuation of a two-end-tethered Fermat yarn is shown as a function of immersion length in liquid. Torsional strokes of up to 44° per mm of actuator length were observed. Like for a polymer that absorbs a liquid or vapor, the immersed yarn swells and this volume change drives torsional actuation.

## 4. Tensile actuation

Tensile actuation as represented by changes in yarn length is generally a more useful actuation mechanism than torsional actuation. Early studies had shown that MWNT yarn tensile actuation activated either electrochemically, electrothermally or by absorption was small in magnitude and not competitive with existing technologies, such as shape memory alloy wires. A significant breakthrough was the discovery that coiled MWNT hybrid yarns could generate very large tensile strains and this work led directly to the discovery of high performance tensile actuators made by twisting and coiling ordinary polymer fibres, such as fishing line and sewing thread.^32^

### 4.1 Optimal configurations for tensile actuation

As for torsional actuation, the performance characteristics of twisted nanofiber yarns as tensile muscles depend upon the testing configuration. Tensile actuation always involves clamping at both ends and usually with the ends tethered to prevent rotation. Normally, the entire length of the yarn is activated. Activating only part of a two-end-tethered sample (as commonly employed for torsional actuators) is complicated in tension. The activated twisted fibre generally contracts in length as a result of the volume expansion. If only one portion of a yarn is activated, then both tensile contraction and torsional rotation occur in the activated portion. The unactivated portion of the yarn is connected to the actuating part and so suffers tensile and torsional strains. The magnitudes of these effects are difficult to determine, as a result of the coupling between yarn twist and yarn length. Twisting a yarn causes a reduction in yarn length and estimates of the stretching–torsion coupling coefficient are of the order ∼200° mm^−1^ % for neat MWNT yarns. Consequently, the retwist of the non-actuating part of a half-activated MWNT yarn could contribute significantly to the overall length contraction. In contrast, the tensile stress imposed on the non-actuating part of a half-activated yarn would partially counter any contraction in the actuating part. Experimental results shown in Fig. 5B highlight the complexity in analysing the tensile actuation of partially activated yarns. Here, the fully immersed (completely activated) two-end-tethered, electrochemically activated yarn gave only slightly larger contraction strains than the same sample tested when only half immersed in electrolyte (partially activated). Because of these complications, it is preferable to conduct tensile actuation tests in the fully activated, two-end-tethered configuration.

**Fig. 11:**
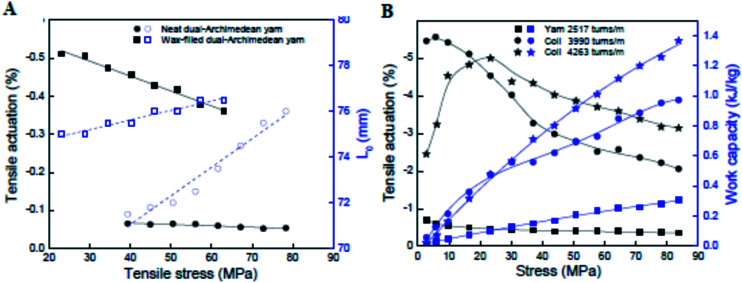
(A) Tensile actuation (left axis) and length (right axis) *versus* applied stress for non-coiled dual-Archimedean yarns having 20 000 ± 500 turns per m of inserted twist and about the same diameter before (17.5 ± 0.5 μm and 16.4 ± 0.9 μm, respectively) and after wax infiltration (18.1 ± 0.9 μm and 16.2 ± 1.1 μm, respectively). The lines are a guide to the eye. (B) The stress dependence of steady-state tensile actuation and contractile work (black and blue data points, respectively) produced by Joule heating (0.189 V cm^−1^) for a 150 μm diameter, dual-Archimedean yarn having different levels of inserted twist. This figure has been reproduced from ref. 32 with permission from AAAS.

Another important testing condition is the amount of tension applied to the yarn. As illustrated in Fig. 11, the tensile actuation strain magnitude decreases with increasing stress applied under isotonic (constant stress) conditions. This decrease in strain magnitude is likely due to a decrease in yarn Young's modulus due to heating so that the modulus shift causes a lengthening in proportion to the applied stress. This length increase partially offsets the heat-induced yarn length contraction. The situation is more complex in coiled yarns [Fig. 11B]. Here the tensile actuation magnitude first increases and then decreases with increasing applied isotonic stress. The initial increase in actuation strain has been linked to the separation of the coils that permits the heat-induced length contraction. When there is little separation between adjacent coils, the coils can come into contact and prevent any further length contraction.

### 4.2 Tensile actuation performance

#### 4.2.1 Electrochemical actuation in twisted MWNT yarns

Prior to the discovery of torsional actuation in MWNT yarns, it had already been established that these same materials generated a surprising lengthwise contraction when electrochemically charged.^33^ Earlier studies on non-twisted MWNT fibres and unoriented CNT sheets had all reported expansions during electrochemical charging. While surprising when first reported, the lengthwise contraction of twisted MWNT yarns is now understood to be a consequence of the helically twisted topology of MWNT bundles in the yarn. Electrochemically driven volume expansion of the yarn causes both untwisting and length contraction with a diameter direction expansion. The theoretical bases of these mechanisms are described in Section 5.

Electrochemically driven twisted yarn tensile actuation strain tends to couple strongly with torsional strokes, as illustrated in Fig. 8. Consequently, the tensile strain increases with increasing applied potential difference from the potential of zero charge and the strain magnitude is affected by the type of electrolyte ions present. The largest tensile strains approach −1% when neat MWNT yarns were electrochemically charged to extreme potentials (+5 V, *versus* Ag/Ag^+^ reference) and when contracting against the applied 88 MPa load, which is equivalent to lifting a mass 185 000 times the mass of the actuating nanotube yarn segment. The peak power generated is 920 W kg^−1^, which is three times higher than the torsional power output obtained during the same charging process.

#### 4.2.2 Thermal tensile actuation in twisted and coiled hybrid MWNT yarns

A surprising discovery was the very large tensile contractions produced by coiled MWNT hybrid yarns. As described in Section 2, the coiled yarns were generated by extreme twist insertion and the presence of solid paraffin wax fixed the coiled shape. As explained in subsequent work on coiled polymer fibres,^32^ the torsional actuation of the yarn from which the coil is made actually drives the change in coil length. Just as stretching a helical spring causes twisting of the spring wire, so too does a heat-induced untwist of the MWNT yarn cause a contraction of the coiled MWNT hybrid yarn.

The effects of wax infiltration and yarn coiling are demonstrated in Fig. 11. Electrothermal heating of neat twisted yarns gave small tensile contractions of −0.05%. Filling these same yarns with paraffin wax caused an order of magnitude increase in tensile strains under the same input stimulus to around −0.5% [Fig. 11A]. A further order of magnitude increase occurred by coiling the wax-filled yarns. As shown in Fig. 11B, the twisted yarns again produced tensile strains of approximately −0.5%. By inserting additional twist to induce coiling, the tensile actuation increased to the range −3% to −5%, depending upon the applied stress. Coiling of the yarns clearly enhances tensile strokes, but coiling also decreases the tensile stiffness in comparison with the same mass of non-coiled yarns of the same yarn diameter. Interestingly, the specific work output of the coiled yarns was 3–4 times higher than that of the equivalent non-coiled yarn [Fig. 11B] despite the reduced stiffness of the former.

**Fig. 12:**
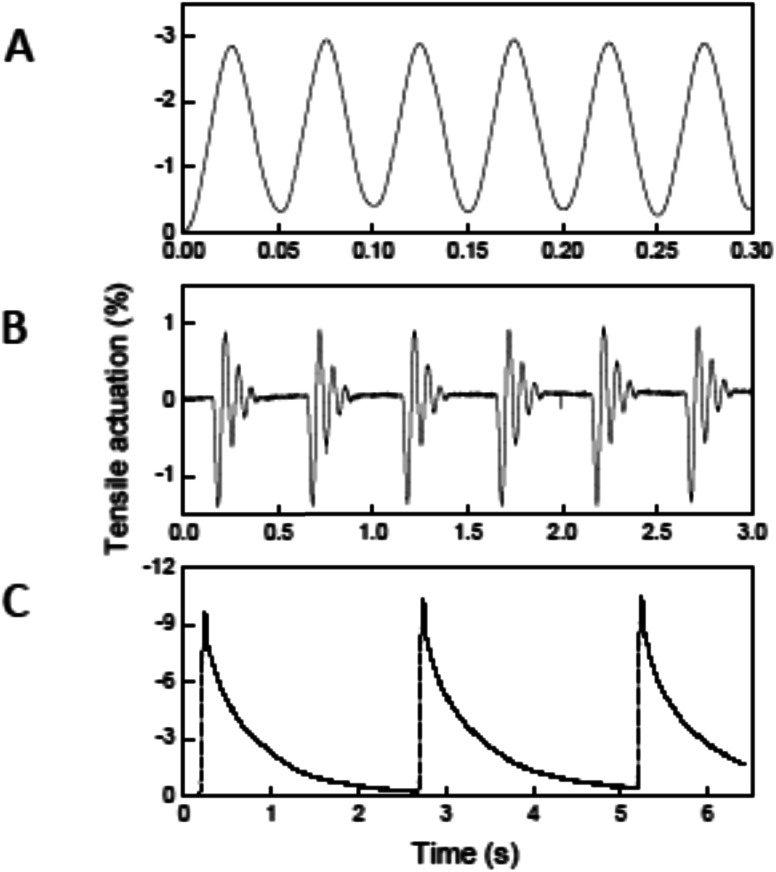
Electro-thermal tensile actuation for two-end-tethered, wax-filled MWNT yarns. (A) Tensile actuation strain *versus* time after 1 400 000 reversible cycles for a 11.5 μm diameter, coiled yarn having ∼25 000 turns per m twist when driven by a 18.3 V cm^−1^, 20 Hz symmetric square wave voltage while lifting a load that provided a 14.3 MPa stress. (B) Tensile actuation for the yarn of (A) with 109 MPa applied tensile stress when driven at a 3% duty cycle by 15 ms, 32 V cm^−1^ square-wave voltage pulses having a period of 500 ms. (C) Tensile strain *versus* time for a 150 μm diameter coiled yarn when supporting a 5.5 MPa tensile stress and driven by a 15 V cm^−1^ square wave having a 50 ms pulse duration and 2.5 s period. This figure has been reproduced from ref. 4 with permission from AAAS.

As for the torsional actuation in hybrid twisted yarns, fast and stable tensile actuation was also observed. Tensile actuation at 1200 cycles per minute and 3% stroke was demonstrated for over 1.4 million cycles [Fig. 12A] using a two-end-tethered, wax-filled, coiled yarn that lifted 17 700 times its own weight.^4^ This high-rate was produced by applying fast voltage pulses to these small diameter yarns (11.5 μm and 20 μm yarn and coil diameters, respectively) so that passive cooling to room temperature occurred in just 25 ms. Applying well-separated 25 ms voltage pulses yielded 1.58% initial contraction [Fig. 12B] and 0.104 kJ kg^−1^ of mechanical energy during this contraction at an average power output of 4.2 kW kg^−1^. The actuator performance of this yarn was further optimized by increasing the applied voltage and mechanical load, while reducing the pulse duration. Fig. 12B shows a series of actuations wherein the yarn lifts 175 000 times its mass in 30 ms when 32 V cm^−1^ is applied for 15 ms. The work during contraction (0.836 kJ kg^−1^) provided a power output of 27.9 kW kg^−1^, which is 85 times the peak output of mammalian skeletal muscles (0.323 kW kg^−1^)^34^ and 30 times the measured power density of previous carbon nanotube muscles.^3^ However, the high applied electrical power reduces cycle life by causing excessive heating and slow paraffin evaporation. Finally, tensile actuations as large as −10% were observed in coiled, wax-filled yarns of higher yarn diameter (150 μm), as shown in Fig. 12C. The higher diameters reduced the passive cooling time and low contractile power density when both heating and cooling times are considered (0.12 kW kg^−1^).

#### 4.2.3 Absorption driven tensile actuation in coiled hybrid MWNT yarns

Solvent absorption has also been used to deliver large stroke torsional actuation in coiled, hybrid MWNT yarns.^35^ These hybrid yarns contained a silicone rubber guest material that could be swollen by exposure to non-polar solvents. High levels of guest incorporation were possible by first preparing a MWNT yarn of low twist and infiltrating with uncured silicone rubber resin. After room temperature vulcanisation of the rubber, the hybrid yarn containing 90–95 wt% rubber was then twisted to induce coiling. Exposing the silicone-MWNT hybrid yarns to various solvents followed by solvent evaporation produced reversible and stable coil contractions and expansions of up to 50% stroke and for over a thousand cycles. The same samples could also be activated electrothermally to produce a stroke of 33% and with a faster response than solvent absorption and desorption. The solvent driven muscles generated a maximum specific work of 1180 J kg^−1^ or 4.4 kW kg^−1^ power output during the contraction part of the cycle. Using a solvent exchange system, the actuators could also be retained in a ‘catch state’ where the immersed actuator maintains its length without requiring any external source of energy. Theoretical upper estimates of energy conversion efficiency were up to 16% for these systems.

## 5. Single helix model of torsional and tensile actuation in twisted yarns

**Fig. 13:**
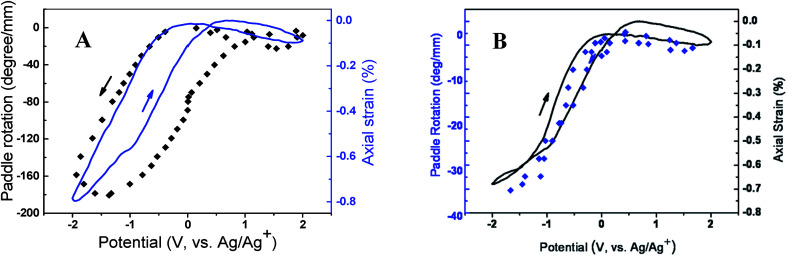
Electrochemical torsional and tensile actuation for two-end-tethered, neat MWNT yarns half immersed in liquid electrolyte. Yarns were either prepared as S twist (A) or Z twist (B). Negative paddle rotations indicate rotation in the untwist direction and negative strains indicate length contraction. This figure has been reproduced from ref. 3 with permission from AAAS.

All the experimental evidence obtained from neat and hybrid MWNT yarns indicates that a volume increase causes simultaneous partial untwist and length contraction. Not unexpectedly, the rotation direction for torsional actuators depended upon the twist direction imparted during yarn spinning. For example, when viewed from the in-air end of a two-end tethered yarn, a paddle attached to the midpoint of a S twist yarn (a right handed yarn) rotated in the clockwise direction when electrochemically charged, while a paddle attached to a Z twist yarn (a left handed yarn) rotated in the counter clockwise direction [Fig. 13]. In both cases the paddle rotation direction corresponded to untwisting of the yarn during charging.

**Fig. 14:**
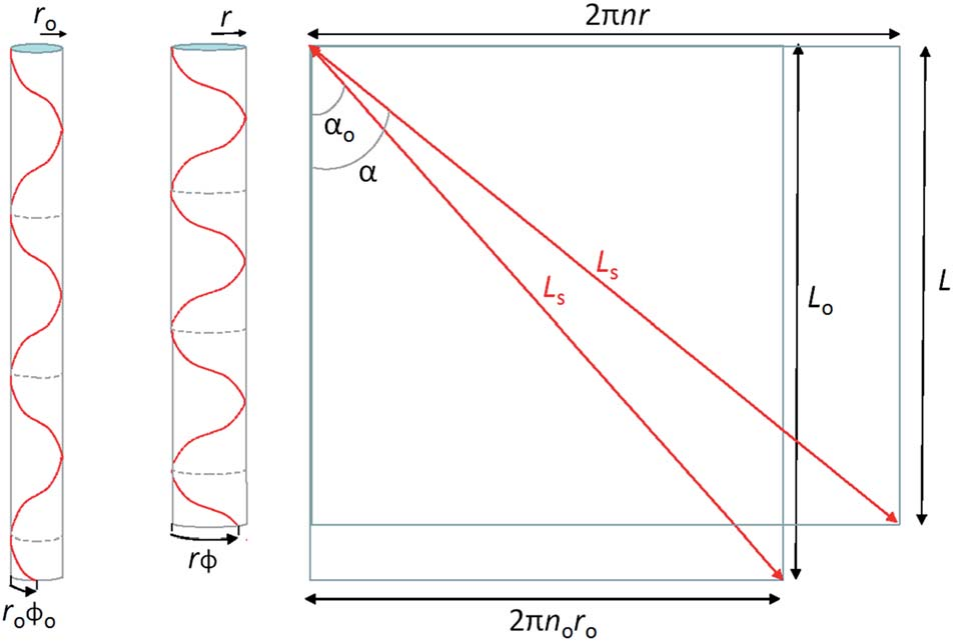
Single helix model for a twist-spun nanotube yarn, where a single helically wound fiber of constant length *L*_s_ forms a cylindrical volume of radius *r*_o_ and length *L*_o_ before actuation and *r* and *L* after actuation (left and middle illustrations). The fiber makes *n*_o_ turns along the cylinder length before actuation and *n* turns after actuation, and the rotation of the fiber bottom end with respect to the top is *ϕ*_o_ before actuation and *ϕ* after actuation. The illustration on the right shows the helically wound fibers on the left unwound to make straight fibers, thereby providing the relationship between the yarn bias angle (*α*), the yarn radius, and the number of turns in the helix. As shown here, cos(*α*) = *L*/*L*_s_ and sin(*α*) = 2*n*π*r*/*L*_s_. This figure has been reproduced from ref. 3 with permission from AAAS.

A simple single helix model provides a reasonable basis to understand the origin of torsional and tensile actuation resulting from volume changes in highly twisted yarns. The model is a major simplification of the real yarn structure that is composed of multiple intertwined helices that vary in twist rate and diameter. However, the single helix analogy offers an analytically simple method to probe how helix volume changes can relate to end torsion and overall length changes, providing qualitative predictions of the yarn mechanical response. Fig. 14 illustrates a single fiber helically wound though *n* rotations at a helix bias angle *α* to enclose a cylinder of length *L* and radius *r*. The fiber length is *L*_s_ and the rotation of the fiber end with respect to its starting point at the top is *ϕ*. The volume enclosed by the helically wound fiber is:

2 *V* = *L*_s_^3^ sin^2^(*α*)cos(*α*)/4π*n*^2^,

since *V* = π*r*^2^*L* and *r* = *L*_s_ sin(*α*)/2π*n* and *L* = *L*_s_ cos(*α*). Alternatively, the cylinder volume can be expressed in terms of fiber length as:

3 *V* = *L*(*L*_s_^2^ − *L*^2^)/4π*n*^2^.

We are interested in examining the dependence of helix rotation on the change in helix volume. Rotation is indicated by a change in the number of turns, with a decrease in *n* indicating untwisting of the helix and an increase in *n* corresponding to an increase in twist. Using eqn (3), the ratio of the number of turns after a volume change to the initial number of turns is:4
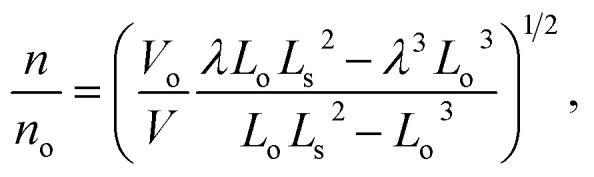
where *V*_o_ and *L*_o_ are for the initial state, *V* and *L* are for the actuated state, and *λ* is the length ratio (*L*/*L*_o_). Eqn (4) and the dependence of the yarn bias angle on inserted twist and yarn diameter provide a means to evaluate the effect of volume change on relative twist (*n*/*n*_o_) and/or relative length (*L*/*L*_o_) of analogous twist yarns. In all the following analyses, the initial length of the fiber that forms the single helix is assumed to be constant.

**Fig. 15:**
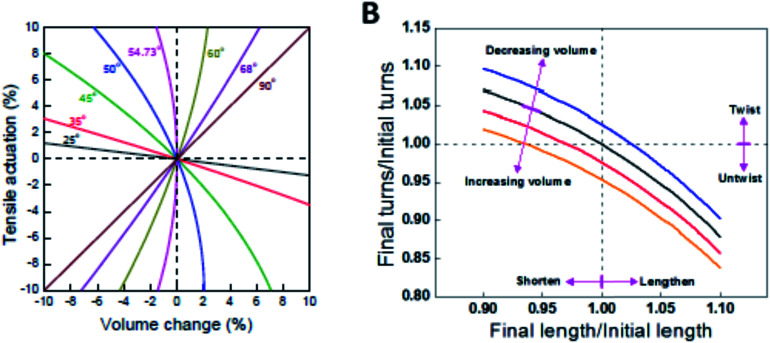
(A) Calculated tensile strain as a function of volume change for single helices having various initial bias angles. The string length is constant and torsional rotation is prohibited. (B) Values of relative twist (*n*/*n*_o_) and relative length (*L*/*L*_o_) that are mutually compatible with the helix model for specified values of an initial helix bias angle of 40°. Blue, black, red, and orange lines are for yarn volume changes of −5%, 0%, 5%, and 10%, respectively. This figure has been reproduced from ref. 3 with permission from AAAS.

Fig. 15A shows the values of relative length (*L*/*L*_o_) that are expected when rotation is prohibited and for specified values of the initial helix bias angle and percent volume change. For low bias angles (less than the “magic angle” 54.73°), increasing yarn volume will cause the yarn to contract in length. For bias angles greater than the magic angle, the yarn will increase in length when the yarn volume is increased if the yarn rotation is prevented. All twisted yarns evaluated to date have starting bias angles of less than 50° and their contraction with volume increase is in agreement with the predictions of the single helix model.

The expected torsional and tensile actuations for three different starting bias angles are illustrated in Fig. 15B. These analyses show that an infinite combination of final length and final twist is geometrically possible for a given volume change. As shown in Fig. 15B, for a starting bias angle of 40° which is typical of MWNT yarns, a volume increase can lead to predicted yarn untwist and lengthening; untwist and shortening; or retwist and shortening. Certainly, if length is fixed the model predicts yarn untwist. Similarly, if rotation is prevented the model predicts a contraction in length. However, it is not yet clear why the simultaneous torsion and tensile actuation produces an untwist and shortening when other combinations are possible, at least in theory.

## 6. Graphene fiber actuators

### 6.1 Fabrication of graphene fiber actuators

**Fig. 16:**
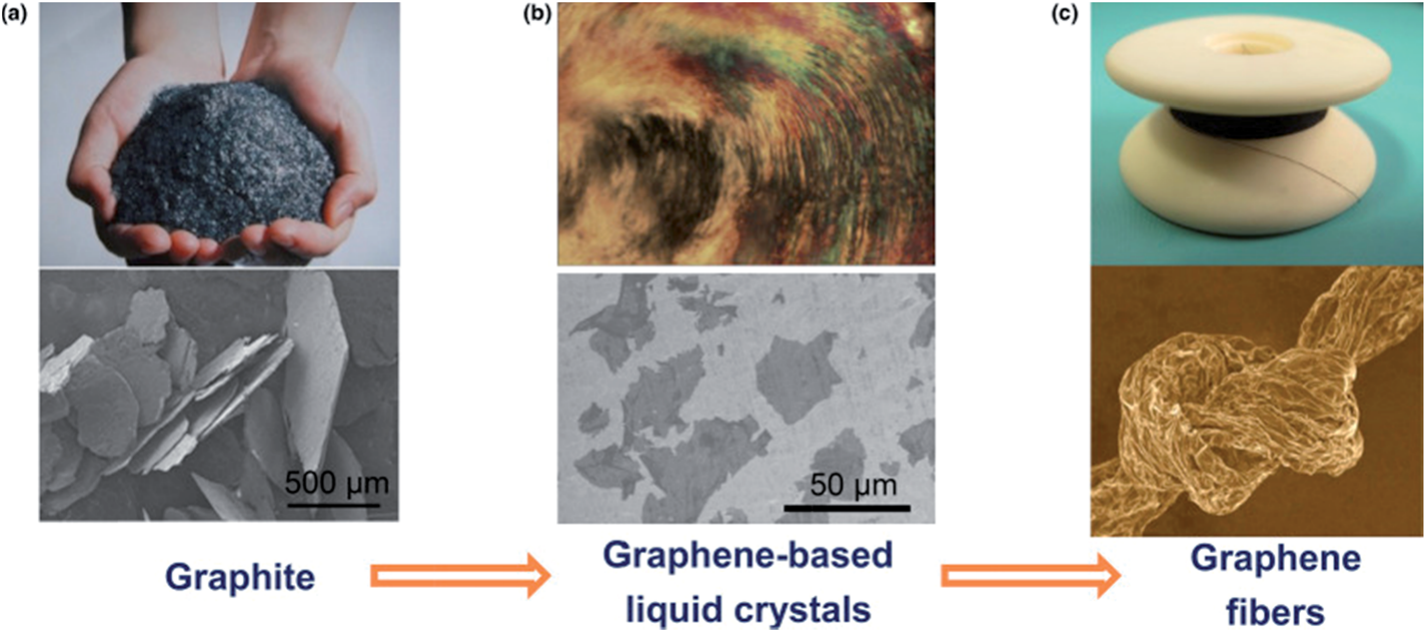
The road from graphite to graphene fibers. In the first step, graphite crystals (a) are exfoliated into individual graphene sheets, usually by chemical modification. The modified graphene sheets form liquid crystals in solvents with orientational or positional order (b). In the second step, wet-spinning assembly is employed to make continuous graphene fibers (c) from these graphene-based liquid crystals, which transform order from the fluid state to order in the solid state. This figure has been reproduced from ref. 36 with permission from Elsevier.

The recent success in assembling graphene sheets into macroscopic fibres has inspired extensive interest in these materials because of the lower cost of graphene fibres compared with CNTs and commercial carbon fibres, and their practical importance for specific applications. The liquid crystalline graphene oxide structure allows for the dispersion of graphene oxide at high enough concentrations suitable for efficient alignment and effective coagulation. Fig. 16 shows the fabrication of graphene fibers from graphite.

**Fig. 17:**
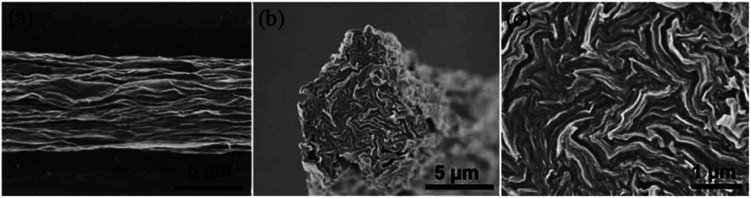
SEM images of the surface (a) and failure ends (b and c) of wet-spun graphene fibers. The fiber surface is not smooth, and there are plenty of dentate-bends in the fiber cross section. This figure has been reproduced from ref. 37 with permission from Wiley-VCH.

Spinning of liquid crystalline (LC) suspensions of large sheet graphene oxide (GO) in water has been recently reported by several research groups. Use of large GO sheets has enabled the use of a wet-spinning route to produce strong fibres by extruding them through a thin nozzle into an appropriate coagulation bath which can be easily converted to electrically conducting graphene fibres by using an appropriate chemical reducing agent. SEM micrographs of the wet-spun graphene fibers are shown in Fig. 17.

**Fig. 18:**
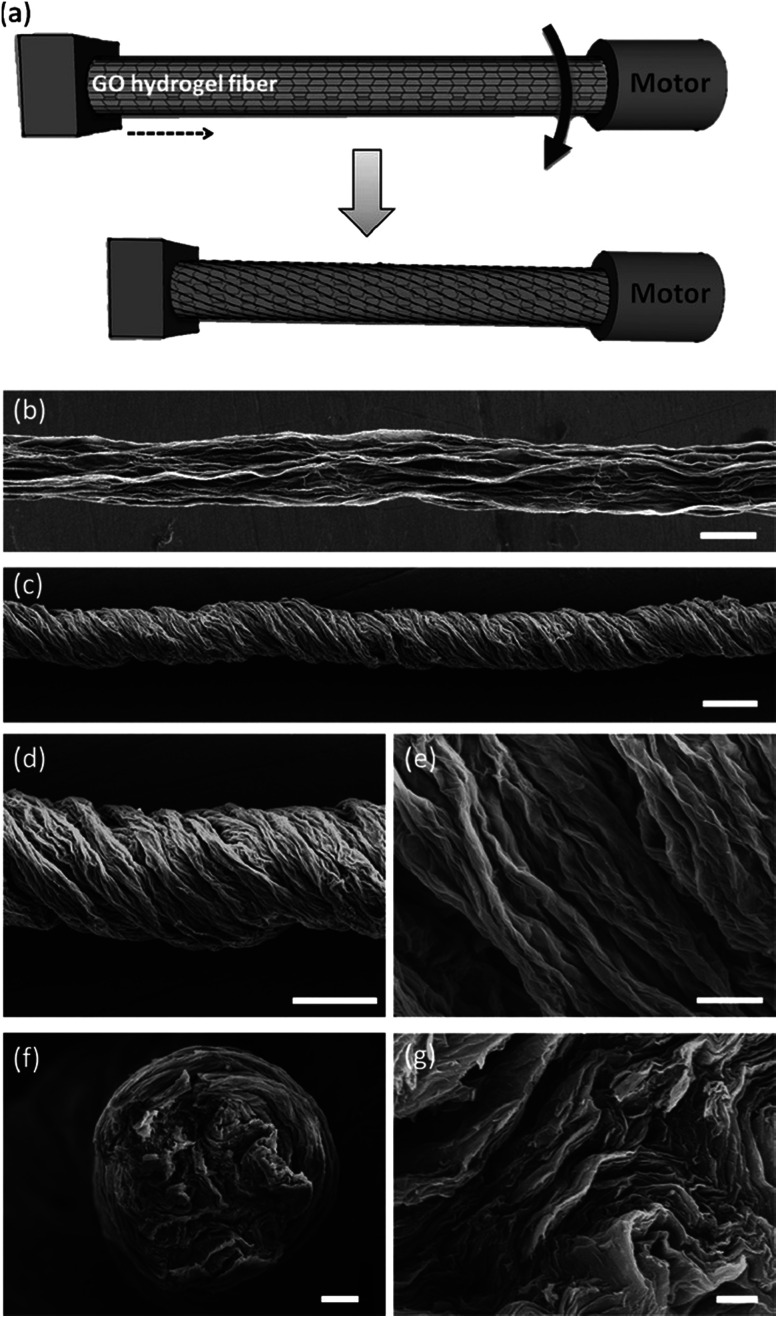
Fabrication and characterization of graphene fiber artificial muscles, (a) scheme of the graphene fiber fabrication; the arrow indicates the direction of rotation; (b and c) SEM images of the directly dried graphene fiber and twisted graphene fiber; (d and e) enlarged view of the twisted graphene fiber and its surface respectively; (f) cross-section of a graphene fiber; (g) enlarged cross-section of the graphene fiber. This figure has been reproduced from ref. 11 with permission from Wiley-VCH.

To develop actuators based on wet-spun graphene fibers, the twisting strategy which has been utilized as a simple and efficient approach for optimizing the strength, flexibility and other characteristics of fiber structures was used to create graphene fiber artificial muscles. As schematically illustrated in Fig. 18 the twisted graphene fiber can be conveniently fabricated by simply twisting the freshly spun graphene fiber along the axis.^11^

### 6.2 Fabrication of the hybrid CNT/graphene fiber actuator

**Fig. 19:**
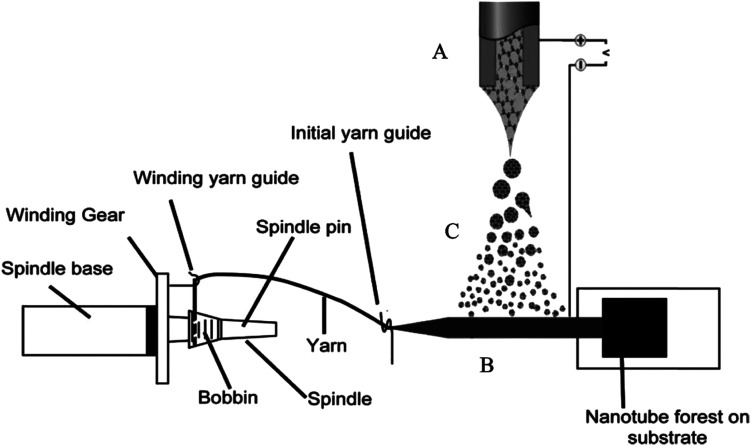
Schematic diagram of a continuously produced hybrid CNT/graphene yarn. (A) Electrospinning setup used for graphene deposition; (B) MWNT sheet drawn from a spinnable forest and employed as the graphene collector; (C) graphene dispersion (electrospray). This figure has been reproduced from ref. 38 with permission from Wiley-VCH.

**Fig. 20:**
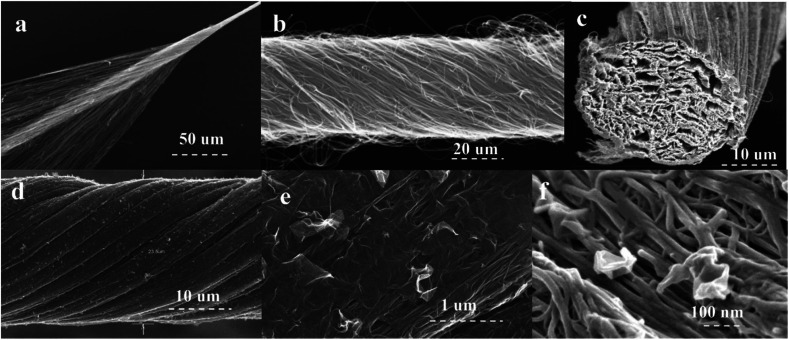
SEM images of (a) the CNT forest during twist insertion to form pristine yarns, (b) the pristine CNT yarn, and the hybrid carbon nanotube–graphene yarn: cross-section (c) at low and (f) high magnification; hybrid yarn surface (d) at low and (e) high magnification. This figure has been reproduced from ref. 38 with permission from Wiley-VCH.

An actuator based on the carbon nanotube and graphene composite fibers has also been reported recently. Foroughi *et al.* reported a novel approach to develop MWNT/graphene nanocomposite fibers by electro-spinning of chemically converted graphene (CCG) within and on the surface of MWNT yarns (Fig. 19).^38^ The CCG dispersion was incorporated into the pre-formed MWNT forest using electrospinning. The composite yarn exhibited improved mechanical, electrical and electrochemical properties as compared with the pristine MWNT material and graphene sheets. SEM images of MWNT and MWNT/graphene fibers are shown in Fig. 20. As can be seen from Fig. 20, graphene nanosheets are present on the surface of highly twisted carbon nanotube fibers.

**Fig. 21:**
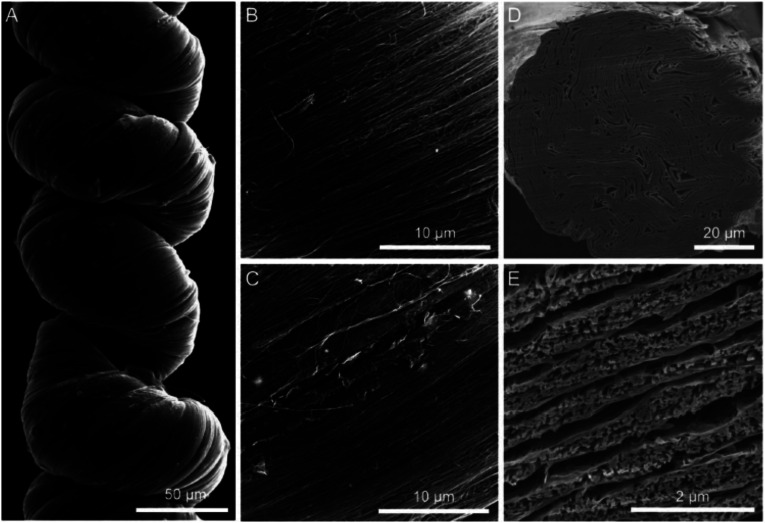
SEM images of a coiled CNT/rGO hybrid yarn containing 40 wt% rGO. (A) Low-magnification SEM image of a coiled CNT/rGO yarn that was prepared by twisting seven layers of 2-cm-wide CNT sheets to a twist density of ≈25 000 turns per m. (B) High-magnification SEM images showing that CNTs were well aligned on the surface of the hybrid yarn and that (C) graphene flakes could be observed at the small cracks on the hybrid yarn. (D) Low-magnification and (E) high-magnification cross-sectional SEM images of the hybrid yarn before coiling, which shows a layered biscrolled structure. This figure has been reproduced from ref. 15 with permission from Wiley-VCH.

More recently, Qiao *et al.*, described a similar approach to develop highly twisted and coiled CNT/graphene fibers.^15^Fig. 21 shows the SEM images of the coiled CNT/graphene fibers.

### 6.3 Moisture-activated torsional graphene fiber actuator

**Fig. 22:**
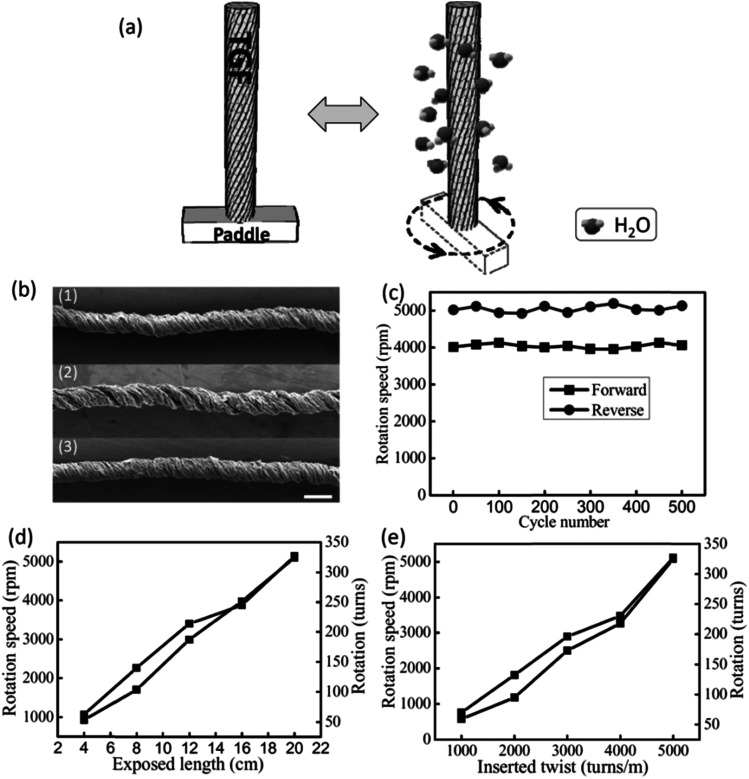
(a) Schematic rotation of a twisted graphene fiber with a paddle at the low (left) and high (right) humidity. When the moisture increases (right), the twisted graphene fiber can drive the paddle rotating fast; then the paddle can reverse to the initial state when the moisture decreases (left). (b) SEM images of the initial twisted graphene fiber at RH = 20% (1), after exposure to a high humidity of 85% (2), and the final state of the twisted graphene fiber as the humidity goes back to the initial RH = 20% (3); scale bar: 100 μm. (c) The durability test of the twisted graphene fiber (5000 turns per m) undergoing repeated RH changes, showing forward (the environmental humidity changed from RH = 20% to 85%) and backward (RH = 85% to 20%) rotation speed *versus* cycle numbers. (d) The rotation speed and rotation numbers *versus* different twisted graphene fiber lengths exposed to RH = 85%. (e) The rotation speed and rotation numbers *versus* twisted graphene fibers with different applied twists of 1000, 2000, 3000, 4000, 5000 turns per m at a length of 20 cm. The environmental humidity changes from RH = 20% to 85%. This figure has been reproduced from ref. 11 with permission from Wiley-VCH.

Highly twisted wet-spun graphene fibers have been used to develop moisture-driven torsional actuators. The twisted graphene fiber with rearranged graphene sheets within the fibers presents large strokes and reversible rotary actuation with a rotation speed of up to 5190 revolutions per minute and a tensile expansion of 4.7% under humidity alternation.^11^ Due to the oxygen-rich functional groups of GO, the relatively fast and reversible expansion/contraction of GO layers will take place through the adsorption and desorption of water molecules, which could accordingly cause a large deformation of 5%. Therefore, the formed helical geometry of GO fibers would enable the reversible torsional rotation under the alternation of humidity (Fig. 22a). As expected, a reversible change of helical angle was observed for the twisted graphene fiber under the variation of humidity (Fig. 3A and B). For a twisted graphene fiber with an initial *α* of 46.2° at a relative humidity (RH) of 20% (Fig. 22b-1), the *α* value decreases to about 42° with exposure to moisture (RH = 85%) (Fig. 22b-2). The decrease of twist degree results from the spontaneously reverse rotation of the twisted graphene fiber due to the adsorption of water molecules and thus the volumetric expansion of the twisted graphene fiber. Once the moisture was removed, the twisted graphene fiber almost recovers its initial configuration (Fig. 22b-3).

The twisted graphene fiber can quickly rotate a paddle when exposed to moisture with a RH of 85% (Fig. 22a), while the fiber performs a 4.7% expansion along the axis. The twisted graphene fiber can completely and rapidly reverse to the initial state as the environmental humidity regresses to the ambient condition (Fig. 22b). The maximum torsional rotation rate was 5190 rotations min^−1^ (543 rad s^−1^) and the whole rotation process consisted of 327 full turns (117 720°). During 500 cycles of reversible rotation, the fiber maintains the rotation forward and backward stably (Fig. 22c). Because the total actuation length is 20 cm for the measured sample, the observed torsional rotation is 588° mm^−1^, which is more than twice the value of the carbon nanotube torsional actuator (250° mm^−1^) powered by electricity,^3^ and thousands of times the values reported for other torsional actuators based on shape-memory alloys, piezoelectric ceramics, and conducting polymers with generated torsional rotations of 0.15° mm^−1^, 0.008° mm^−1^, and 0.01° mm^−1^, respectively.^11^

### 6.4 Hybrid CNT/graphene fiber actuator

**Fig. 23:**
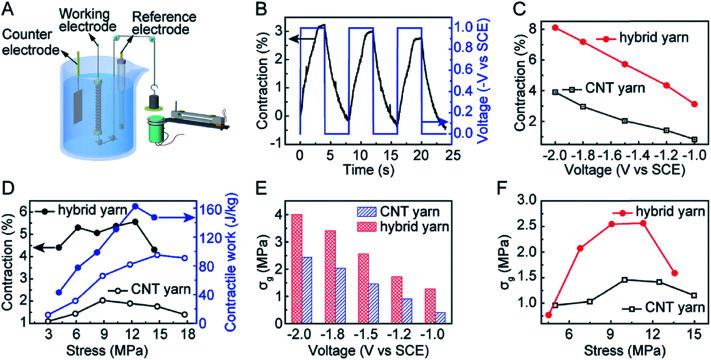
Characterization of the electrochemical actuation of coiled neat CNT muscles and coiled hybrid yarn muscles containing 40 wt% rGO, when a 0.125 Hz square-wave potential was applied. (A) Schematic diagram of a home-made apparatus for characterizing isobaric actuation. A yarn muscle was the working electrode, a saturated calomel electrolyte was the reference electrode, and a CNT film (which had been preactivated in 0.1 m HNO_3_) was the counter electrode. Muscle contraction was measured using a contactless electromagnetic sensor. The stress generated during isometric muscle actuation was measured using a cantilever beam microstress sensor (Fig. 8) after an initial bias stress was applied to avoid initial intercoil contact. (B) The time dependence of applied potential (*V*, right axis) and resulting muscle contraction strain (left axis) of the hybrid yarn muscle while lifting a 12 MPa load when a 0.125 Hz square-wave potential of −1 V was applied. (C) Contractile stroke as a function of applied 0.125 Hz square-wave potential for a CNT yarn muscle and a hybrid yarn muscle when an applied isobaric load of 12 MPa was applied. (D) The stress dependence of the contractile stroke (left axis) and corresponding contractile work capacity (right axis) for CNT yarn muscle and CNT/rGO hybrid yarn muscle when a 0.125 Hz square-wave potential of −1.5 V was applied. (E) The generated isometric contractile stress *versus* applied 0.125 Hz square-wave potential for the hybrid yarn muscle and the neat CNT yarn muscle when an initial bias stress of 10 MPa was applied to avoid initial intercoil contact. (F) The dependence of isometrically generated contractile stress on the initial applied initial bias stress for a −1.5 V, 0.125 Hz square-wave potential. This figure has been reproduced from ref. 15 with permission from Wiley-VCH.

Artificial muscles based on hybrid carbon nanotubes/graphene yarns have been developed using a three-electrode electrochemical system, which is shown in Fig. 23A.

As can be seen from Fig. 23B, the coiled CNT/graphene hybrid yarn actuator shows 3.1% contraction. In fact, the coiled hybrid yarn muscle provided higher contractile strokes than the coiled neat yarn muscle in the investigated potential range (Fig. 23C). These comparative results indicate the effectiveness of graphene in improving actuator performance. An almost linear dependence of the contractile stroke on the applied potential was observed for both the coiled neat CNT yarn and the coiled hybrid yarn muscles (Fig. 23C).

Fig. 23D plots the contractile stroke (left axis) and the generated contractile work per cycle (right axis) as a function of applied stress for the neat CNT yarn muscle and the CNT/graphene hybrid yarn muscle. In addition, Fig. 23E shows the generated contractile stress *versus* applied square wave potential for the hybrid yarn muscle and the neat CNT yarn muscle when the bias stress is 10 MPa. The contractile stress increased with the increase of applied potential and the maximum stress reached 4.0 MPa at a potential of −2 V. The sum of this stress and the bias stress (14 MPa) is about 40 times the contractile stress of natural skeletal muscles. The dependence of isometric contractile stress on the applied bias stress when a 0.125 Hz, −1.5 V square-wave potential was applied is shown in Fig. 23F. The hybrid yarn muscle achieved a maximum contractile stress of 2.56 MPa, which is nearly twice the maximum contractile stress of the neat CNT muscle (which was obtained for a bias stress of about 10 MPa).

## 7. Sheath-run artificial muscles

**Fig. 24:**
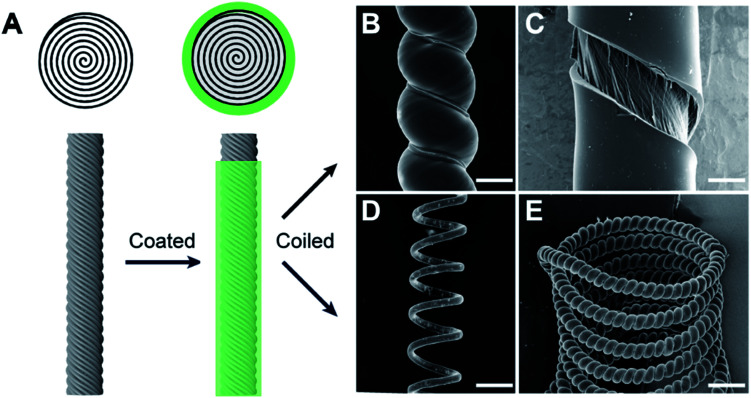
Muscle fabrication and structures for torsional and tensile actuation. (A) Schematic lateral and cross-sectional views of a twisted CNT yarn and a SRAM, which was made by coating the twisted CNT yarn with a polymer sheath. SEM micrographs of PEO-SO_3_@CNT muscles showing (B) a SRAM made by self-coiling of a sheath-coated twisted yarn, (C) the surface of a twisted SRAM that was broken by untwisting in liquid N_2_, showing a distinct boundary between the sheath polymer and the CNT core, (D) a mandrel-coiled twisted SRAM, and (E) a SRAM that was self-coiled and then mandrel-coiled. The scale bars for (B)–(E) are 35, 15, 200, and 200 μm, respectively. This figure has been reproduced from ref. 9 with permission from AAAS.

**Fig. 25:**
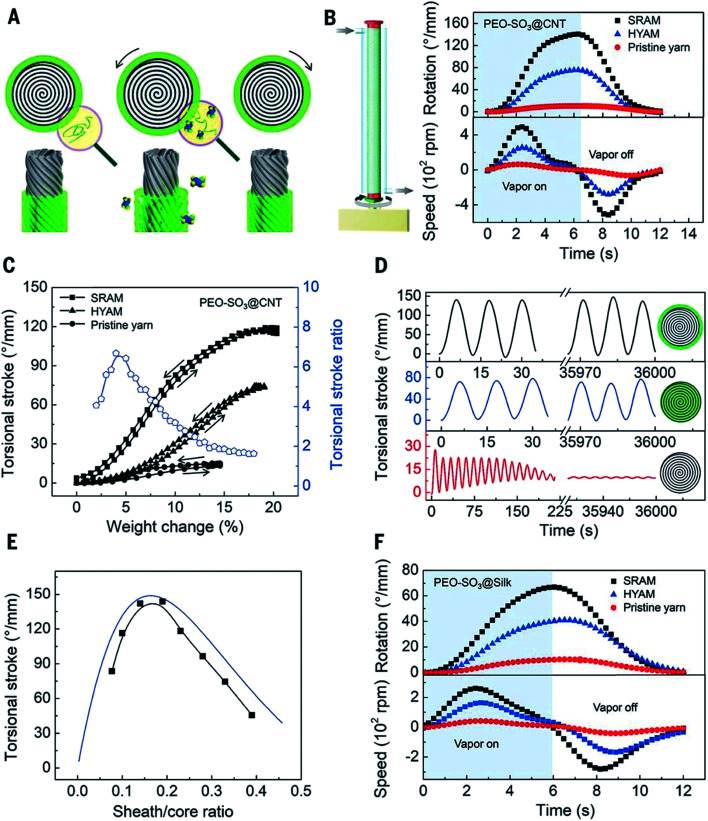
Torsional actuation of twisted PEO-SO_3_ SRAMs and HYAMs driven by ethanol-saturated dry air. (A) Illustration of a vapor-driven PEO-SO_3_ SRAM before vapor exposure (left) and during vapor sorption (middle) and desorption (right), which cause yarn untwist and retwist, respectively. (B) Illustration of vapor delivery to a one-end-tethered muscle (left) and plots of the time dependence of the torsional stroke and rotation speed for one sorption/desorption cycle for a PEO-SO_3_@CNT SRAM and HYAM and for a pristine CNT yarn (right). The 41 μm-diameter pristine yarn, with 72 turns per cm of twist, was used for fabricating the 45 μm-diameter SRAM and 50 μm-diameter HYAM which contained a weight ratio of PEO-SO_3_ to CNT of 0.53. (C) The dependence of the equilibrium torsional stroke (black squares) on muscle weight changes due to ethanol absorption and desorption for the muscles of (B), and the corresponding ratio of SRAM to HYAM strokes (blue circles, during ethanol absorption). (D) Torsional stroke *versus* cycle number for the muscles of (B). (E) The observed (black squares) and predicted (blue line) dependence of the torsional stroke on the sheath/core ratio for PEO-SO_3_@CNT SRAMs. (F) Torsional stroke and rotation speed *vs.* time for a sorption/desorption cycle of a PEO-SO_3_@silk SRAM and HYAM and a silk yarn. The 56 μm-diameter silk yarn (with 5.7 turns per cm of twist) was used for fabricating the 89 μm-diameter SRAM and the 91 μm-diameter HYAM, which both weighed 0.48 mg cm^−1^ and contained the same weight ratio of PEO-SO_3_ to silk (0.27). This figure has been reproduced from ref. 9 with permission from AAAS.

**Fig. 26:**
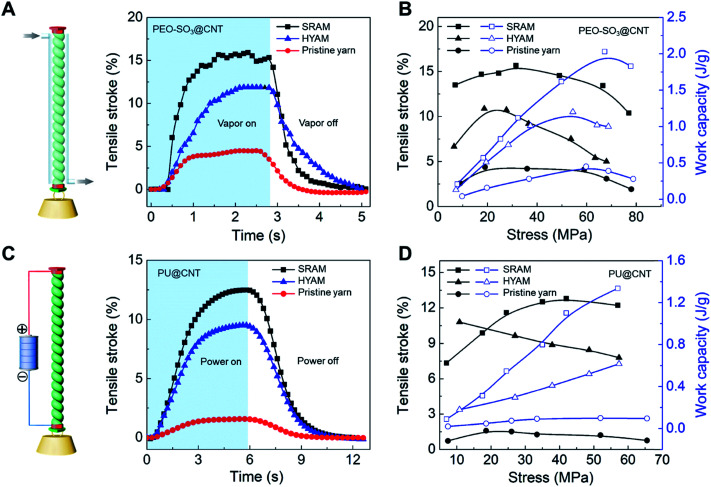
Isobaric tensile actuation of self-coiled, sorption-powered and electrothermally powered SRAMs, HYAMs, and pristine CNT yarns. (A) Tensile stroke *vs.* time for a PEO-SO_3_@CNT SRAM and HYAM and a pristine yarn when actuated by ethanol absorption using the configuration shown on the left and a stress of 33 MPa. Sorption was from a near-equilibrium ethanol concentration in dry air and desorption was by dynamic pumping. Before coiling, the diameters of the PEO-SO_3_@CNT SRAM and HYAM and the pristine yarn were 43, 47, and 38 μm, respectively. (B) Tensile stroke and contractile work capacity *vs.* applied stress for the sorption-actuated muscles of (A). (C) The time dependence of the tensile stroke for a PU@CNT SRAM and HYAM and a pristine CNT yarn when electrothermally actuated under 42 MPa stress using 0.25 W cm^−1^ power, which provided temperatures of 85, 93, 97 °C, respectively. The device structure is shown on the left. Before coiling, the diameters of the PU@CNT SRAM and HYAM and the pristine yarn were 65, 71, and 51 μm, respectively. (D) Tensile stroke and contractile work capacity *vs.* applied stress for the electrothermally actuated yarns in (C). This figure has been reproduced from ref. 9 with permission from AAAS.

Most recently we have reported a new generation of hybrid carbon nanotube yarn artificial muscles, known as sheath-run artificial muscles (SRAMs), featuring a sheath around a coiled or twisted yarn, which contracts, or actuates, when heated, and returns to its initial state when cooled. The outside sheath absorbs energy and drives actuation of the muscle. SRAMs have an alternative muscle topology that provides higher performance, wherein the guest that drives actuation is a sheath on a twisted or coiled core that can be an inexpensive yarn (Fig. 24). This topology change from guest-filled to sheath-run artificial muscles increases the maximum contractile work capacity by factors between 1.85 and 2.15 for muscles that are driven electrothermally, electrochemically, or by vapor-absorption (Fig. 25). The muscles are made from carbon nanotube yarns and/or common natural and man-made fibres, such as cotton, silk, wool and nylon. A sheath-run coiled electrochemical muscle provides a maximum average contractile power density (2.17 W g^−1^), which is 44 times that of natural muscle and 4.43 times that of the alternative coiled electrochemical carbon nanotube yarn muscle (Fig. 26).

## 8. Applications

**Fig. 27:**
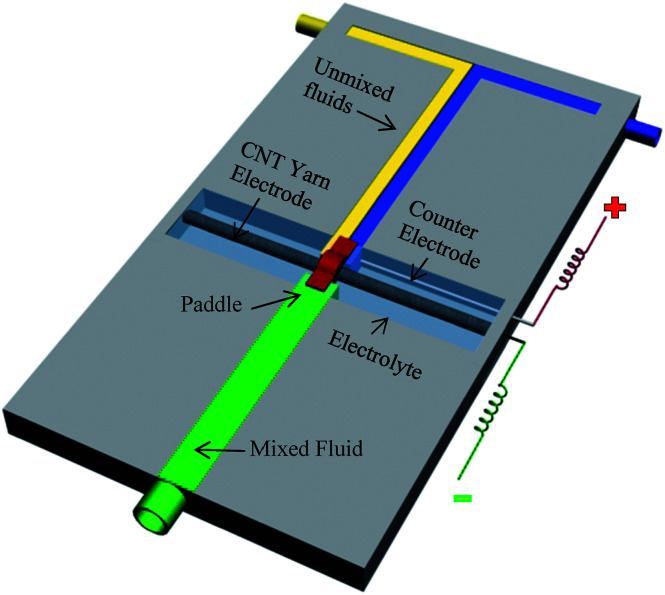
Illustration of a prototype fluidic mixer where incoming unmixed streams (water containing blue and yellow food dye) were mixed by a paddle attached to a MWNT yarn suspended transversely and half immersed in electrolyte. The right hand side cross channel contained the immersed part of the yarn and an auxiliary platinum wire electrode. The left hand side cross channel was unfilled. The paddle was torsionally oscillated by applying a square wave voltage of 0 V or −3 V at 1 Hz between the MWNT yarn and the auxiliary electrode. The electrolyte was 0.2 M TBAPF_6_ in acetonitrile. This figure has been reproduced from ref. 3 with permission from AAAS.

An active mixer for fluidic chips was fabricated to demonstrate an application of nanotube-yarn-based torsional actuators. Though the widths of the fabricated fluidic channels were large, the easily obtainable yarn diameters (down to at least 4 μm) are compatible with microfluidic devices having much narrower channels. The schematic of the fluidic mixer is illustrated in Fig. 27. The prototype was made by machining 3 mm deep × 3 mm wide channels in a solid sheet of poly(methylmethacrylate) to make a ‘T’ junction. Two additional cross channels, each 30 mm long, were cut perpendicular to the 120 mm long main flow channel. A 65 mm length of 15 μm diameter nanotube yarn was positioned horizontally and fixed at the far ends of each cross channel. One end of the yarn was attached to a length of 50 μm diameter platinum wire that provided electrical connection. The cross channels and the main flow channel were not connected and the nanotube yarn passed over the top of the short barriers separating each cross channel from the main flow channel. One perpendicular cross channel was filled with 0.2 M TBAPF_6_ in acetonitrile and the other cross channel was unfilled. The yarn was, therefore, half immersed in electrolyte and half in air. The electrolyte channel was also fitted with a 250 μm diameter platinum wire that acted as a counter electrode. No reference electrode was used. A small piece of polyester film (3 mm × 1 mm) was glued to the yarn and positioned in the center of the flow channel to serve as a paddle. This paddle was reversibly rotated forwards and backwards by applying a 0 V to −3 V square-wave voltage at 1 Hz between the torsionally actuated nanotube yarn electrode and the counter electrode. This voltage cycle produced a paddle rotation of up to 180°. Water colored with food dye was pumped in through the two inlet ports using a syringe pump (KD Scientific) operating at 50 mL h^−1^ flow rate. The blue and yellow flows did not mix due to laminar flow along the full length of the main flow channel. The presence of the paddle (when still) in the flow stream did not promote mixing between the blue and yellow layers. However, complete mixing could be rapidly achieved by torsional actuation of the paddle in the path of the flowing liquids.

**Fig. 28:**
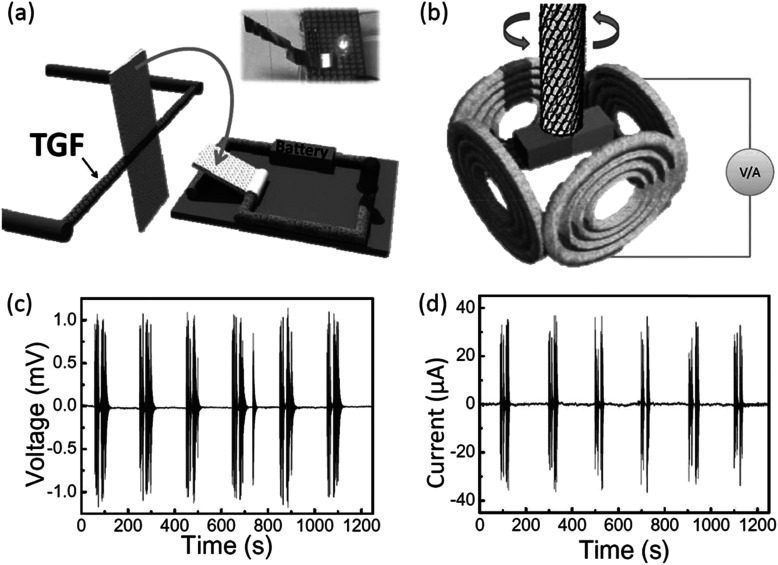
The scheme of the designed humidity switch (a) and the alternating current generator (b) based on the humidity-responsive twisted graphene fibers. In the switch (a), the twisted graphene fiber in response to moisture (*e.g.*, RH = 85%) can rotate a paddle to press on the metal plate, as pointed out by the arrow, so that the electric circle powered by the battery will turn on the LED, as shown in the inset photo. The generator (b) contains four copper coils around the twisted graphene fiber with a magnet. When the environmental humidity changes, the twisted graphene fiber can reversibly rotate the magnet within the surrounding copper coils to generate electricity, (c and d) open-circuit voltage and short-circuit current of the generator tested under humidity changes between 20% and 85%. This figure has been reproduced from ref. 11 with permission from Wiley-VCH.

Furthermore, the excellent moisture-driven torsional actuation of the twisted graphene fiber enabled us to fabricate a new type of humidity switch. As shown in Fig. 28a, the device included two parts. The left part is the control section with a paddle attached on the twisted graphene fiber (65 mm in length). The right part is a simple circuit including a light emitting diode (LED), a battery and an aluminium sheet which can be pressed on/off through the contact or disconnection of two ends of the circuit. When the environmental humidity increases, the twisted graphene fiber will drive the paddle rotation in the direction of the arrow shown in Fig. 28a, which will trigger the switch and turn on the LED (Fig. 28a, inset). Upon removal of the moisture, the twisted graphene fiber will pull the paddle to the original position and release the press on the aluminium pad, which hence switches off the light. In addition the unique twisted graphene fiber with sensitivity in response to the moisture provides a chance to develop a new type of humidity-triggered electric generator, which will produce power using mechanical work induced by the variation of ambient moisture. A 20 cm length twisted graphene fiber was made with 5000 turns per m holding a magnet bar at one end; this bar was located at the center of several copper coils (Fig. 28b). When the whole device was exposed to an environment with varied humidity, the twisted graphene fiber could drive the magnet reversible rotation and so lead to the generation of electricity in the copper coils. This generator produced an open-circuit voltage of up to 1 mV (Fig. 28c), and a short-circuit current of up to 40 μA (Fig. 28d).

**Fig. 29:**
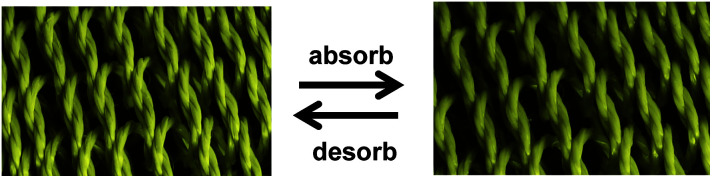
Application of moisture-responsive, sheath-run artificial muscles (SRAMs) for comfort-adjusting textiles. Photographs showing the porosity increase of a knitted SRAM textile when exposed to water. This reversible porosity change enables evaporation of sweat. This figure has been reproduced from ref. 9 with permission from AAAS.

Water-responsive twisted sheath-run artificial muscles (SRAMs) were also used to fabricate knitted textiles that respond to the presence of perspiration by opening pores. Fig. 29 shows the reversible actuation that occurs when the knitted SRAM textile was sprayed with water. Absorption of water caused the hole area of the textile to increase from 42.2% of the total textile area before water exposure to 60.8% after water exposure. This increase in porosity resulted from yarn untwist to produce increased writhing. This increased writhing caused the textile to shear like a collapsing wine rack, thereby increasing porosity and decreasing the length in the warp direction by 18.6%, while maintaining the length in the weft direction.^9^

## 9. Conclusions and future work

The demonstration of twisted and coiled multi-walled carbon nanotube and graphene yarns has provided novel high-performance torsional and tensile actuators. The first generation of torsional carbon nanotube yarn actuators was operated by electrochemical double-layer charge injection, like for CNT supercapacitors. The extreme twist insertion in these carbon nanotube yarns, combined with the volume expansion occurring during electrochemical charging, generated giant torsional actuation strokes of up to 250° per mm with speeds of 600 revolutions per minute. The experimentally derived peak power output per weight of electrochemically actuated yarn was 71 W kg^−1^.^3^

The practical limitations of the electrochemical CNT yarn actuators were overcome in the second generation hybrid materials that used an incorporated guest material to generate the required volume change. Such systems could operate in air or in liquids and did not require the electrolyte, additional electrodes and packaging needed for the electrochemical systems. Hybrid yarns could be conveniently controlled using electrothermal means by taking advantage of the highly conducting CNT yarn host. The maximum torsional strokes of ∼60° mm^−1^ for these electrothermally actuated CNT yarns containing the paraffin wax guest were smaller than the largest strokes obtained electrochemically. However, rotation speeds were considerably higher (up to 11 500 revolutions per minute) and the systems operated stably for more than one million cycles.^4^

Tensile actuation was also demonstrated in all twisted CNT yarns, while the stroke, work and power outputs were dramatically increased by incorporation of the yarn guest and by over-twisting the yarn to form coils. Fast, highly reversible tensile actuation was demonstrated for paraffin-wax filled yarns. Strokes of up to 10% were observed in coiled, wax-filled yarns. For small diameter yarns, actuation at 1200 cycles per minute giving 3% stroke was demonstrated for over 1.4 million cycles. In the tensile mode, power outputs during contraction reached 27.9 kW kg^−1^, or 85 times that of skeletal muscle.^3,4^

A new generation of hybrid carbon nanotube yarns “*Sheath-Run Artificial Muscles*” enabled the maximum contractile work capacity to be increased by factors between 1.85 and 2.15 for muscles that are driven electrothermally, electrochemically, or by vapor-absorption. A sheath-run coiled electrochemical muscle provides a maximum average contractile power density (2.17 W g^−1^), which is 44 times that for natural muscle and 4.43 times that for the alternative coiled electrochemical carbon nanotube yarn muscle.^9^

In addition, artificial muscles based on the twisted graphene fiber were developed that showed the maximum torsional rotation rate of 5190 rotations min^−1^ (543 rad s^−1^) and the whole rotation process consisted of 327 full turns (117 720°).

These performance characteristics make CNT and graphene twisted yarn muscles attractive for high value applications requiring small weights of CNTs or graphene yarns, since CNTs and graphene yarns are presently expensive. While impressive performance has been demonstrated to date, further work is needed to improve the energy conversion efficiency. At present, the highest realized efficiency for the conversion of electrical energy to mechanical energy is ∼0.5% for CNT hybrid yarns.

## Conflicts of interest

There are no conflicts to declare.

## Supplementary Material

## References

[cit1] Baughman R. H. (1999). *et al.*, Carbon Nanotube Actuators. Science.

[cit2] De Volder M. F. (2013). *et al.*, Carbon nanotubes: present and future commercial applications. Science.

[cit3] Foroughi J. (2011). *et al.*, Torsional Carbon Nanotube Artificial Muscles. Science.

[cit4] Lima M. D. (2012). *et al.*, Electrically, Chemically, and Photonically Powered Torsional and Tensile Actuation of Hybrid Carbon Nanotube Yarn Muscles. Science.

[cit5] Gu X. (2016). *et al.*, Hydro-actuation of hybrid carbon nanotube yarn muscles. Nanoscale.

[cit6] Sun Y. (2018). *et al.*, Water-responsive helical graphene-oxide fibers incorporating a continuous carbon nanotube network. Carbon.

[cit7] Song Y. (2018). *et al.*, Hierarchical carbon nanotube composite yarn muscles. Nanoscale.

[cit8] Mirvakili S. M., Hunter I. W. (2018). Artificial Muscles: Mechanisms, Applications, and Challenges. Adv. Mater..

[cit9] Mu J. (2019). *et al.*, Sheath-run artificial muscles. Science.

[cit10] Kim H. (2018). *et al.*, Thermally Responsive Torsional and Tensile Fiber Actuator Based on Graphene Oxide. ACS Appl. Mater. Interfaces.

[cit11] Cheng H. (2014). *et al.*, Moisture-Activated Torsional Graphene-Fiber Motor. Adv. Mater..

[cit12] Cheng H. (2013). *et al.*, Graphene Fibers with Predetermined Deformation as Moisture-Triggered Actuators and Robots. Angew. Chem., Int. Ed..

[cit13] Cheng H. (2014). *et al.*, Graphene fiber: a new material platform for unique applications. NPG Asia Mater..

[cit14] Zhao Y. (2013). *et al.*, Stimulus-responsive graphene systems towards actuator applications. Energy Environ. Sci..

[cit15] Qiao J. (2018). *et al.*, Large-Stroke Electrochemical Carbon Nanotube/Graphene Hybrid Yarn Muscles. Small.

[cit16] Jin K. (2018). *et al.*, Self-plied and twist-stable carbon nanotube yarn artificial muscles driven by organic solvent adsorption. Nanoscale.

[cit17] Mirvakili S. M., Hunter I. W. (2017). Fast Torsional Artificial Muscles from NiTi Twisted Yarns. ACS Appl. Mater. Interfaces.

[cit18] Mirvakili S. M., Pazukha A., Sikkema W., Sinclair C. W., Spinks G. M., Baughman R. H., Madden J. D. (2013). Niobium Nanowire Yarns and their Application as Artificial Muscles. Adv. Funct. Mater..

[cit19] Zhang M., Atkinson K. R., Baughman R. H. (2004). Multifunctional Carbon Nanotube Yarns by Downsizing an Ancient Technology. Science.

[cit20] Zhang M. (2005). *et al.*, Strong, Transparent, Multifunctional, Carbon Nanotube Sheets. Science.

[cit21] Foroughi J. (2012). *et al.*, Preparation and characterization of hybrid conducting polymer-carbon nanotube yarn. Nanoscale.

[cit22] Lima M. r. D. (2011). *et al.*, Biscrolling Nanotube Sheets and Functional Guests into Yarns. Science.

[cit23] Lee J. A. (2014). *et al.*, All-Solid-State Carbon Nanotube Torsional and Tensile Artificial Muscles. Nano Lett..

[cit24] Chun K., Hyeong Kim S., Kyoon Shin M. (2014). *et al.*, Hybrid carbon nanotube yarn artificial muscle inspired by spider dragline silk. Nat. Commun..

[cit25] Keefe A. C., Carman G. P. (2000). Thermo-mechanical characterization of shape memory alloy torque tube actuators. Smart Mater. Struct..

[cit26] Kim J., Kang B. (2001). Performance test and improvement of piezoelectric torsional actuators. Smart Mater. Struct..

[cit27] Mirvakili S. M., Hunter I. W. (2018). Artificial Muscles: Mechanisms, Applications, and Challenges. Adv. Mater..

[cit28] Ruch P. W., Kotz R., Wokaun A. (2009). Electrochemical characterization of single-walled carbon nanotubes for electrochemical double layer capacitors using non-aqueous electrolyte. Electrochim. Acta.

[cit29] Ue M., Murakami A., Nakamura S. (2002). A Convenient Method to Estimate Ion Size for Electrolyte Materials Design. J. Electrochem. Soc..

[cit30] Madden J. D. (2007). Mobile Robots: Motor Challenges and Materials Solutions. Science.

[cit31] Mirvakili S. M. (2013). *et al.*, Niobium Nanowire Yarns and their Application as Artificial Muscles. Adv. Funct. Mater..

[cit32] Haines C. S. (2014). *et al.*, Artificial Muscles from Fishing Line and Sewing Thread. Science.

[cit33] Mirfakhrai T. (2007). *et al.*, Electrochemical actuation of carbon nanotube yarns. Smart Mater. Struct..

[cit34] Josephson R. K. (1993). Contraction Dynamics and Power Output of Skeletal Muscle. Annu. Rev. Physiol..

[cit35] Liu Z. F. (2015). *et al.*, Hierarchically buckled sheath-core fibers for superelastic electronics, sensors, and muscles. Science.

[cit36] Xu Z., Gao C. (2015). Graphene fiber: a new trend in carbon fibers. Mater. Today.

[cit37] Meng F. (2015). *et al.*, Graphene-Based Fibers: A Review. Adv. Mater..

[cit38] Foroughi J. (2014). *et al.*, Highly Conductive Carbon Nanotube-Graphene Hybrid Yarn. Adv. Funct. Mater..

